# Investigation of the antimycobacterial activity of African medicinal plants combined with chemometric analysis to identify potential leads

**DOI:** 10.1038/s41598-024-65369-7

**Published:** 2024-06-25

**Authors:** Phanankosi Moyo, Michael Ofori, Olusola S. Bodede, Madelien Wooding, Ndivhuwo Kevin Khorommbi, Lyndy J. McGaw, Cynthia A. Danquah, Vinesh J. Maharaj

**Affiliations:** 1 Biodiscovery Center, Department of Chemistry, University of Pretoria, Hatfield, Private Bag X 20, Pretoria, 0028 South Africa; 2 Department of Pharmacology, Faculty of Pharmacy and Pharmaceutical Sciences, College of Health Sciences, Kwame Nkrumah University of Science and Technology, PMB, Kumasi, Ghana; 3 Department of Pharmaceutical Sciences, Dr Hilla Limann Technical University, Wa, Ghana; 4 Phytomedicine Programme, Department of Paraclinical Sciences, Faculty of Veterinary Science, University of Pretoria, Onderstepoort, Private Bag X04, Pretoria, 0110 South Africa

**Keywords:** *Mycobacterium tuberculosis*, Tuberculosis, Natural products, Plants, Metabolomics, Antimicrobial drug resistance, Drug discovery, Microbiology, Diseases, Chemistry

## Abstract

The emergence of drug-resistant *Mycobacterium tuberculosis* strains is a threat to global health necessitating the discovery of novel chemotherapeutic agents. Natural products drug discovery, which previously led to the discovery of rifamycins, is a valuable approach in this endeavor. Against this backdrop, we set out to investigate the in vitro antimycobacterial properties of medicinal plants from Ghana and South Africa, evaluating 36 extracts and their 252 corresponding solid phase extraction (SPE) generated fractions primarily against the non-pathogenic *Mycobacterium smegmatis* and *Mycobacterium aurum* species. The most potent fraction was further evaluated in vitro against infectious *M. tuberculosis* strain. *Crinum asiaticum* (bulb) (Amaryllidaceae) emerged as the most potent plant species with specific fractions showing exceptional, near equipotent activity against the non-pathogenic *Mycobacterium* species (0.39 µg/ml ≤ MIC ≤ 25 µg/ml) with one fraction being moderately active (MIC = 32.6 µg/ml) against *M. tuberculosis*. Metabolomic analysis led to the identification of eight compounds predicted to be active against *M. smegmatis* and *M. aurum*. In conclusion, from our comprehensive study, we generated data which provided an insight into the antimycobacterial properties of Ghanaian and South African plants. Future work will be focused on the isolation and evaluation of the compounds predicted to be active.

## Introduction

Tuberculosis, an ancient infectious bacterial disease mainly caused by *Mycobacterium tuberculosis*, remains a major global health conundrum with one third of the world latently infected^1^. Globally, according to the World Health Organisation (WHO), 10.6 million new cases of tuberculosis were reported in 2022^2^. Unfortunately, 1.3 million fatalities caused by this respiratory infectious disease were recorded in this period, making tuberculosis one of the leading causes of mortality in the world^2^. The WHO regions with the highest burden of this disease in 2022 were South-East Asia (46%), Africa (23%) and the Western Pacific (18%)^2^. The WHO African region was particularly affected by tuberculosis with the greatest number of representatives, *ca.* 47%, amongst the 30 high-burden tuberculosis countries in 2022. Moreover, three African countries, including South Africa, were amongst the top eight countries with the highest incidence rates of tuberculosis in the world^2^.

The fight against tuberculosis is currently being incapacitated by the emergence and spread of drug-resistant *M. tuberculosis* strains. In 2022 there were 410 000 incident cases of multidrug-resistant (MDR) and rifampicin-resistant tuberculosis^2^. Drug-resistant tuberculosis is often immensely challenging to cure, requiring an extended period and yet having low treatment success rates^3^. Furthermore, compared to drug-susceptible tuberculosis, MDR and extremely drug-resistant tuberculosis (XDR) are more expensive to treat. For example, in the United States of America it costs, per patient, US$ 17,000 to treat the conventional drug-susceptible tuberculosis while MDR and XDR cost US$ 134 000 and US$ 430 000, respectively^4^. An additional challenge with drug-resistant tuberculosis is that the second-line treatment regimen used for its treatment has severe adverse effects to patients including permanent hearing loss^4^. South Africa is one of the most burdened by this drug-resistant tuberculosis phenomenon. The prevalence of MDR tuberculosis in South Africa has previously been found to be 3.4% and 7% amongst new and retreatment cases of tuberculosis, respectively^5^. The WHO recommended a first-line treatment regimen for tuberculosis consisting of a rifamycin, isoniazid, pyrazinamide, and ethambutol. In their comprehensive study carried out between 2012 and 2014, Ismail et al*.*^6^ found prevalence of resistance to rifampicin, isoniazid, pyrazinamide and ethambutol to be 4.6%, > 5%, 44.7% and 59.1% amongst MDR tuberculosis cases, respectively^6^. This is a precarious challenge posing a significant threat to global health which demands urgent attention. One way of addressing this is by the expeditious discovery and development of effective, affordable, and safe novel antimycobacterial chemotypes.

Natural products have previously served as an indispensable source of effective novel pharmaceutical agents including those used for the treatment of tuberculosis^7^. Microbial derived natural products have been the most crucial in this regard^8^. The most prolific class of microbial derived antibiotics in the battle against tuberculosis have been rifamycins, including rifampicin and rifapentine^8^. Rifamycins were originally isolated from the bacterium *Amycolatopsis rifamycinica*^9^. The rifamycins are an integral component of the first-line treatment regimen for tuberculosis^10^. Other noteworthy microbial derived tuberculosis antibiotics include the aminoglycoside kanamycin, originally isolated from *Streptomyces kanamyceticus*, and its derivative amikacin^8^. Both kanamycin and amikacin are, unfortunately, ototoxic^11^. Streptomycin, first isolated from *Streptomyces griseus*, was the first ever aminoglycoside to have been discovered. It was also the first ever antibiotic that was used for the treatment of tuberculosis, and currently constitutes the second-line regimen artillery used for treatment of MDR tuberculosis^10,12^. While spectinomycin, originally isolated from *S. spectabilis*, has marginal activity against *M. tuberculosis*, its semisynthetic derivatives, the spectinamides, have shown remarkable potency against both the drug-susceptible and MDR tuberculosis in vitro and in vivo^13,14^.

Plants have played a low-key role in Western medicine for the treatment of tuberculosis. However, it’s worth noting that pyrazinamide is an analogue of nicotinamide, a vitamin which occurs across different natural sources including vegetables^15^. Plants have been used in Eastern medicine for the treatment of tuberculosis, particularly in China, for thousands of years^16^. Different compounds including baicalin, and tuberostemonine, have been identified as active ingredients in plants^16,17^. Currently, some Traditional Chinese Medicinal (TCM) herbal remedies serve as complementary medicines co-administered with conventional treatment regimens for tuberculosis. Some of these TCM decoctions have been clinically proven to be efficacious in treating tuberculosis (Reported in Zhang et al.^16^). The African populace has likewise for decades been using traditional medicines for the treatment of tuberculosis^18,19^, including communities in Ghana and South Africa^20–22^. However, challenges associated with handling of the highly infectious *M. tuberculosis* have limited the antimycobacterial activity evaluation of most traditional medicinal plants. Airborne infectious agents such as *M. tuberculosis* can only be handled safely in Biosafety Level III facilities, which very few institutions have^23^. This challenge in handling the bacterium has also made it difficult to isolate the active compounds using the commonly adopted classical bioassay-guided fractionation process. Bioassay-guided fractionation demands several steps, during which fractions and sub-fractions need to be screened against *M. tuberculosis*. Nonetheless, attempts to investigate several plants have provided interesting leads, including *Crinum asiaticum* and *Artemisia afra*^24,25^*.* However, active ingredients against *Mycobacterium* in both plants have not been identified.

To accelerate the discovery of new antimycobacterial leads from African plant species, in the current extensive collaborative investigation, we screened *ca*. 290 extracts and fractions for activity against two non-pathogenic *M. tuberculosis* surrogate strains, *Mycobacterium smegmatis* and *Mycobacterium aurum*. Using metabolomics techniques, we tentatively identified the bioactive natural compounds. We further assessed the activity of these putative compounds using a classification machine learning model.

## Results and discussion

### Plant material collection, extraction, and solid phase extraction (SPE) fractionation

A total of 31 different medicinal plant species were evaluated in the study (Table 1 and Supplementary File Table S1). These plant species were selected for evaluation based on either their traditional medicinal use, or that of related species, for the treatment of tuberculosis^20^ and other bacterial diseases. The Asteraceae plant family was the most represented with seven species (Table 1). It was closely followed by the Amaryllidaceae which had five different species with most of them assigned to the genus *Crinum*. The Lamiaceae plant family had three representatives closely followed by Meliaceae, Zingiberaceae, and Hypericaceae which each had two representative species. The most investigated plant parts were the leaves. Bulbs were used in the case of *Crinum* spp. while whole plants, flowers and stems were utilised for some plant species. Each plant species provided one extract and seven fractions. Exceptions were *Hedychium flavescens*, *Cyrtanthus mackenii*, *Tulbaghia simmleri*, *Crinum jagus* and *Crinum asiaticum* which each provided two different extracts (of different parts of the plant) and their 14 respective fractions (Table 1). From the plant extraction and high-throughput pre-fractionation process, a total of 36 crude extracts and their corresponding 252 solid phase extraction (SPE) generated fractions (7 fractions per extract) were attained. All extracts and fractions were completely dissolved in 100% dimethyl sulphoxide (DMSO) at a stock concentration of 20 mg/mL. These stock solutions were stored in 96 deep-well plates covered with aluminum plates at 4 °C in a fridge prior to being subjected to in vitro biological evaluation.

**Table 1:**
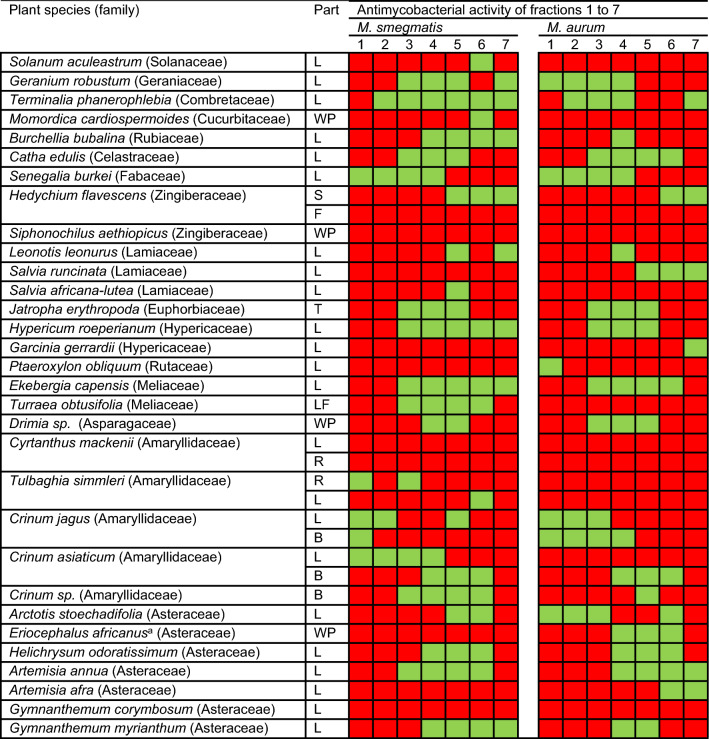
Plant species investigated in vitro for their activity against *M. smegmatis* and *M. aurum*.

1 to 7 are fractions F1 to F7. Green shaded boxes indicate active fractions (MIC ≤ 200 µg/mL). Red shaded boxes indicate inactive fractions (MIC > 200 µg/mL). The extraction and SPE fractionation process led to the realisation of 36 extracts and their 252 SPE fractions. Two different parts of the plants *H. flavescens*, *C. mackenii*, *T. simleri*, *C. jagus* and *C. asiactum* were used in the study.

*L* leaves, *B* bulbs, *WP* whole plant, *R* roots, *S* stem, *F* flower, *LF* leaf and flowers, and *T* tuber.

^a^*Eriocephalus africanus var. paniculatus*. Plant species names and families were verified on the World Flora Online database (http://www.worldfloraonline.org/).

### Antimycobacterial properties of extracts and their fractions

Having acquired the crude extracts of plants and their SPE fractions, the next step was to assess their in vitro antimycobacterial activity. This assessment was carried out using the high-throughput spot culture growth inhibition assay (HT-SPOTi) method^26^. The Middlebrook 7H10 agar culture medium supplemented with 0.5% glycerol and 10% (v/v) oleic albumin dextrose and catalase (OADC) was used for the assay. The activity of the extracts and fractions was evaluated against *M. smegmatis* and *M. aurum*. Both strains, *M. smegmatis* and *M. aurum*, have the advantage of being non-pathogenic and hence can be handled regularly with relative ease in a BSL-2 biosafety cabinet. Furthermore, they are fast growing and most importantly have a drug susceptibility profile which closely resembles that of the infectious *M. tuberculosis* strain^8^. Rifamycin served as a positive control drug for the inhibition of proliferation of *M. smegmatis* and *M. aurum* with MIC values of 7.813 and 3.907 µg/mL, respectively, consistent with that reported in other studies.

The results showed that most species demonstrated some level of potency against either one of the two *Mycobacterium* strains or both (Table 1). *Mycobacterium smegmatis* was the most susceptible *Mycobacterium* species with 79 fractions being active (MIC ≤ 200 µg/mL), representing a 31% hit rate (calculated by dividing the total number of active fractions by the total number of fractions screened against the bacterial species, i.e., 252). Sixty-seven fractions were active against *M. aurum* with a calculated hit rate of 27%. The SPE generated fractions F4 and F5 had the greatest number of actives totaling 32 and 31, respectively, against both *M. smegmatis* and *M. aurum* while F1 and F2 had the least number of actives totaling 10 and 11, respectively. The plant species which had the greatest number of active fractions against both *M. smegmatis* and *M. aurum*, were *Terminalia phanerophlebia* and *Ekebergia capensis*, each with a total of 10 and 9 active fractions, respectively, against both strains. They were closely followed by *Geranium robustum*, *Senegalia burkei, Hypericum roeperianum*, and *Artemsia annua* each with eight active fractions. *H. flavescens* (flowers), *Siphonochilus aethiopicus*, *C. mackenii* (leaves and roots), and *Gymnanthemum corymbosum* did not have any active fractions against either of the two *Mycobacterium* strains (Table 1). There was no plant family which distinctly emerged as the most promising in having the highest proportion of active fractions. However, the Lamiaceae emerged as the least attractive, having the fewest number of active fractions per plant species part evaluated, i.e., an average of 2.3 active fractions (calculated by dividing the total number of active fractions in the family with the total number of extracts evaluated in the family).

Further examination of the potency of the plant species highlighted *C. asiaticum* (bulbs) to be the most active as six of its fractions were highly active (MIC values ≤ 30 µg/mL) against both *M. smegmatis* and *M. aurum* (Table 2). Fraction 5 of *C. asiaticum* emerged as the most active, being equipotent against both *Mycobacterium* strains with a remarkable MIC of 0.39 µg/mL. Other notable highly active SPE fractions against *M. aurum* were those of *G. robustum* (Fractions 1, 2 and 3), *H. flavescens* (stems) (Fraction 6), *Helichrysum odoratissimum* (Fractions 5 and 6), and *Gymnanthemum myrianthum* (Fractions 4 and 5) (Table 2). It was interesting to note that despite having highly active extracts against *M. aurum* (MIC = 12.5 µg/mL), SPE-fractions of *H. flavescens* were all inactive. In contrast to this, SPE-fractions of *G. robustum*, *G. myrianthum* and *C. asiaticum* showed marked activity despite the extract being inactive (MIC > 200 µg/mL) (Table 2). Having emerged as the most active sample, Fraction 5 of *C. asiaticum* and its parent extract were prioritised for further evaluation against the infectious *M. tuberculosis* H37RvMA strain.

**Table 2:**
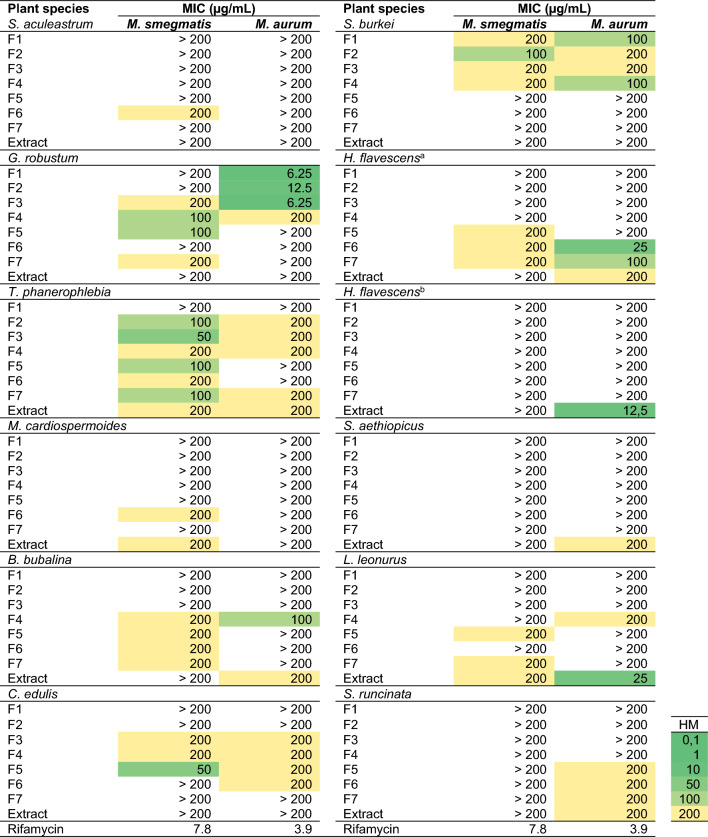

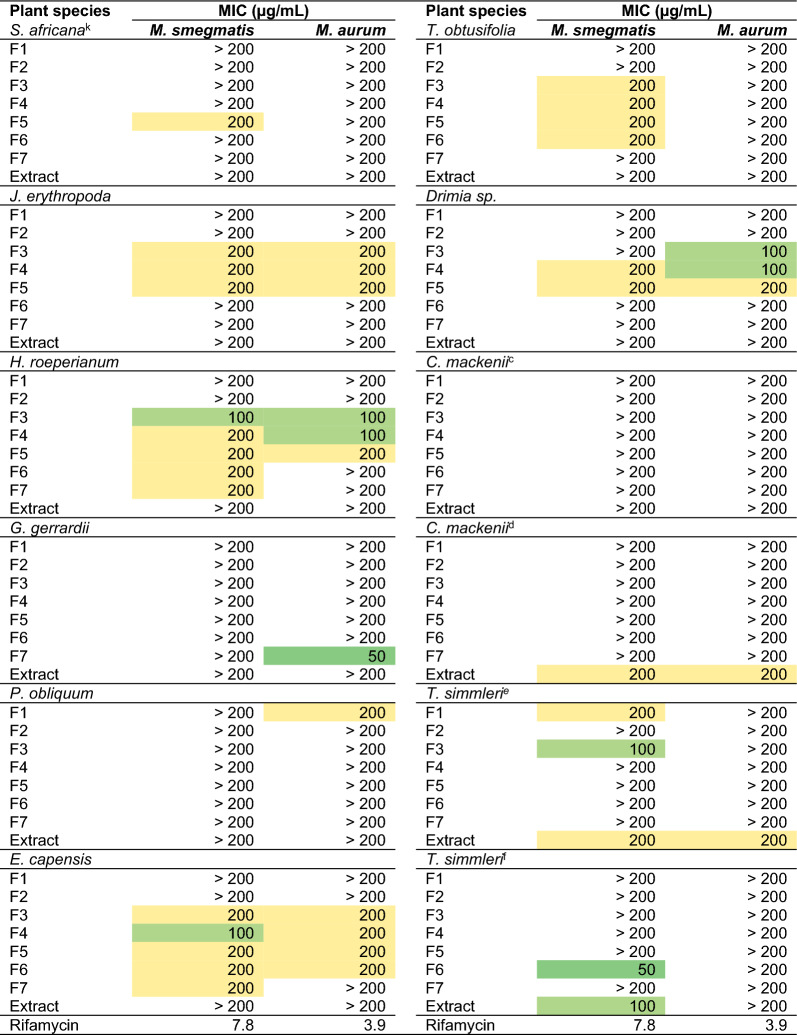

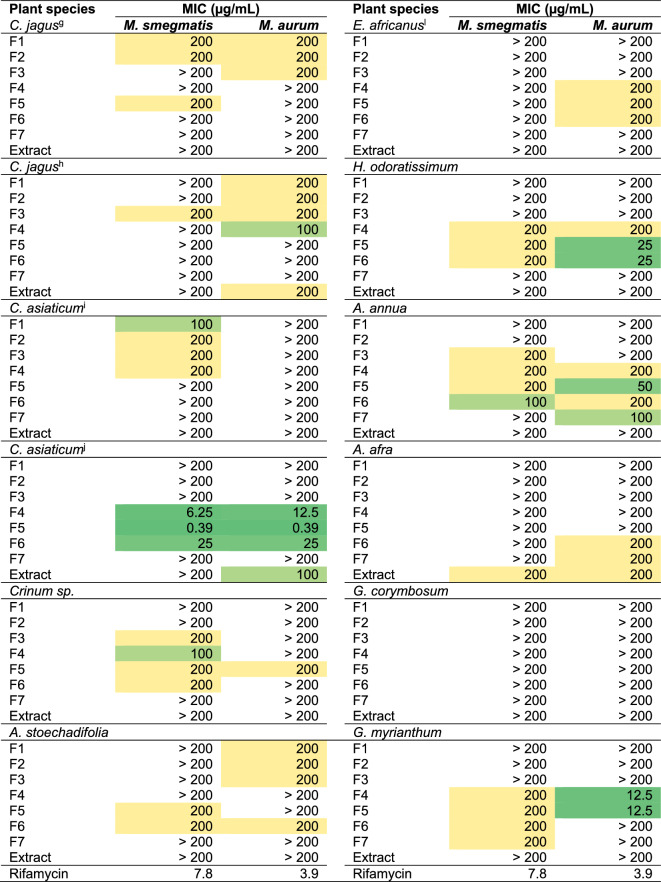
Activity of extracts and fractions against *M. smegmatis* and *M. aurum*.

*HM* heat map showing MIC from low values (dark green) to medium (light green) high values (yellow), *MIC* minimum inhibitory concentration. The Middlebrook 7H10 culture medium supplemented with 0.5% glycerol and 10% (v/v) oleic albumin dextrose and catalase was used for the assay.

^a^Stems, ^b^flowers, ^c^leaves, ^d^roots, ^e^roots, ^f^leaves, ^g^leaves, ^h^bulbs, ^i^leaves, ^j^bulbs, ^k^*S. Africana-lutea*, ^l^*Eriocephalus africanus var. paniculatus.*

The in vitro activity of the crude extract of *C. asiaticum* and its SPE-fraction 5 (F5) were phenotypically assessed against the infectious *M. tuberculosis* H37RvMA strain at the Holistic Drug Discovery and Development Centre (H3D), University of Cape Town, South Africa^27^. The microplate Alamar blue assay was used to determine the MIC of the extract and its fraction^28^. Cognisant of the influence of different media supplements on the activity of compounds in the test samples, the assay was carried out in three different culture mediums (Table 3)^29^. Moxifloxacin and isoniazid served as positive controls with both standard drugs showing expected MIC values (Table 3) consistent with previous studies^30^.

**Table 3:** Activity of extracts and SPE fraction 5 (F5) against *M. tuberculosis.*

*C. asiaticum*	Media	MIC (µg/mL)	Controls	Media	MIC (µg/mL)
Extract	ADC-GLU-TW	> 125.00	Moxifloxacin	ADC-GLU-TW	0.077
	ADC-GLU-TX	> 125.00		ADC-GLU-TX	0.075
	CAS-GLU-TX	> 125.00		CAS-GLU-TX	0.074
F5	ADC-GLU-TW	> 104.37	Isoniazid	ADC-GLU-TW	0.27
	ADC-GLU-TX	> 104.37		ADC-GLU-TX	0.27
	CAS-GLU-TX	32.6		CAS-GLU-TX	0.12

Media used: ADC-GLU-TW: Middlebrook 7H9 media (Difco) supplemented with 0.2% glucose, Middlebrook albumin-dextrose-catalase (ADC) enrichment (Difco) and 0.05% Tween 80. ADC-GLU-TX: Middlebrook 7H9 media (Difco) supplemented with 0.2% glucose, Middlebrook albumin-dextrose-catalase (ADC) enrichment (Difco) and 0.05% Tyloxapol. CAS-GLU-TX: Middlebrook 7H9 media (Difco) supplemented with 0.4% glucose, 0.03% Casitone (Gibco Bacto) and 0.05% Tyloxapol.

Encouragingly, fraction F5 demonstrated moderate activity against *M. tuberculosis* with an average MIC of 32.6 µg/mL (Table 3). This activity was only observed in the serum free medium 7H9_CAS_GLU_TX (Table 3). However, this level of activity for fraction F5 was observed to be *ca*. 100-fold less than that measured against *M. smegmatis* and *M. aurum*. Nevertheless, this activity was regarded as promising, warranting further continued investigation of *C. asiaticum* to identify the active ingredients.

### Metabolomic analysis to predict active compounds in *C. asiaticum*

Having shown its potency against the three different *Mycobacterium* strains, we sought to obtain insight as to which compounds of *C. asiaticum* (bulb) could be active antimycobacterial constituents. Towards this goal, we first classified fractions F4 to F6 as active and the rest as inactive based on their MIC values (≤ 30 µg/mL) against *M. smegmatis* and *M. aurum*. We employed a more robust procedure, namely chemometrics analysis of active and inactive fractions of *C. asiaticum*^31,32^. In this component of the study, for each fraction we acquired their UPLC-HRMS (ESI^+^) data in triplicate. The acquired mass spectrometry data was first pre-processed using Waters MakerLynx XS^®^ resulting in a data matrix file. This file was imported as a Microsoft Excel sheet which was used as an input for multivariate analysis on SIMCA^®^-P.

**Figure 1:**
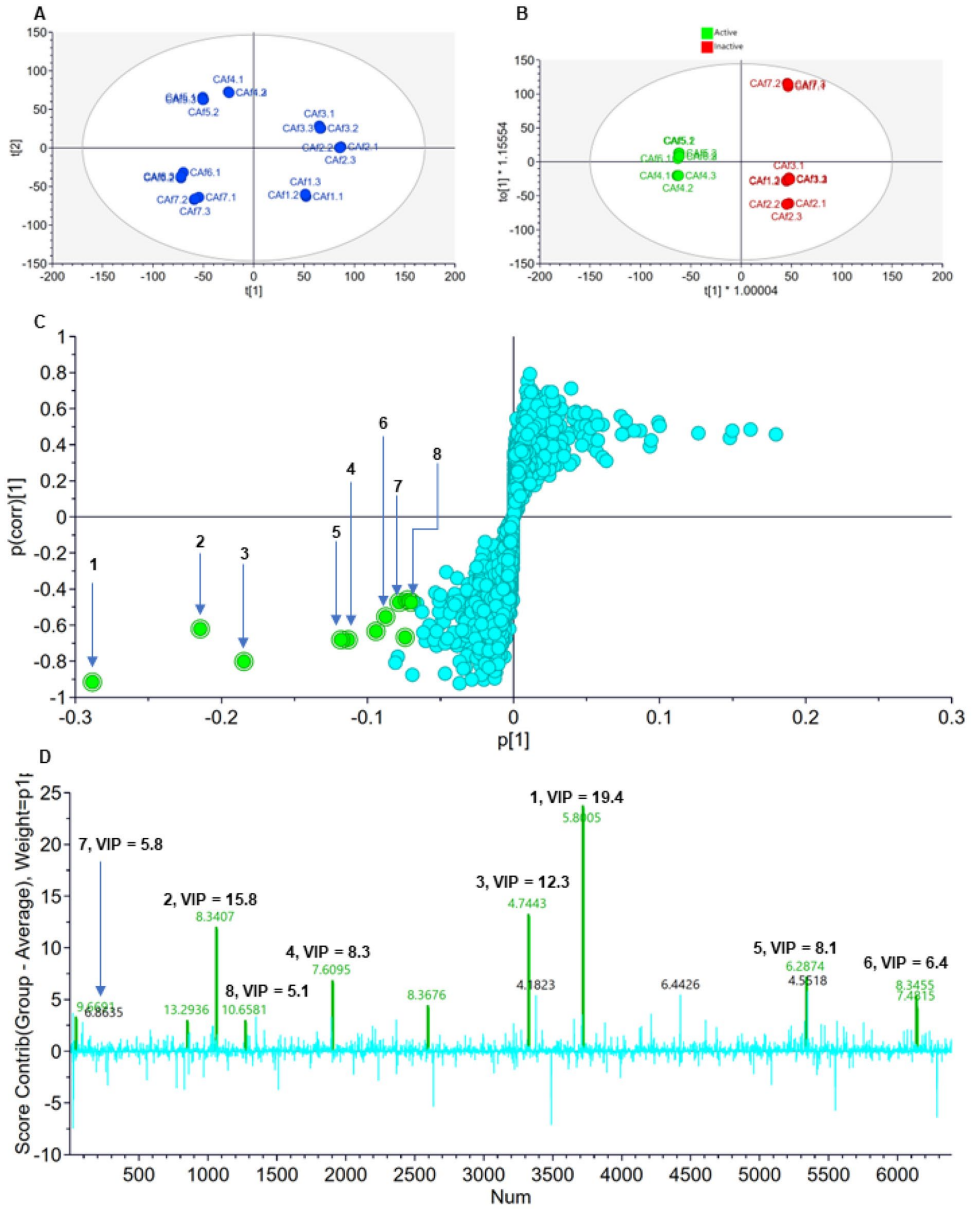
Multivariate analysis of UPLC-MS/MS data of *C. asiaticum* fractions using PCA and OPLS-DA models to identify the potentially active compounds. Unsupervised (**a**) PCA scores plots, (**b**) OPLS-DA scores plots, (**c**) OPLS-DA loadings S-plot and (**d**) contribution plot. Compounds projecting above 0 on the contribution plot are positively associated with activity while those below 0 are negatively correlated to activity. VIP is the variable importance in projection score which is computed for the OPLS-DA model. It provides an estimate on the importance of the respective variable. For each fraction UPLC-MS/MS data was acquired in triplicate, i.e., fraction F1.1 to F1.3 (CAf1.1 to CAf1.3) up to F7.1 to F7.3 (CAf7.1 to CAf7.3). The bottom left quadrant has discriminating features positively associated with activity against *M. smegmatis* and *M. aurum*. (**1**) dihydronarcissidine, (**2**) 3,4-dimethoxyhydrocinnamic acid, (**3**) ambellin, (**4**) acetylcaranine, (**5**) 6-(2-(5-Hydroxy-1,4-dioxo-1,4-dihydronaphthalen-2-yloxy)acetamido)hexanoic acid, (**6**) 3,4-dihydroxyphenylglycol, (**7**) 3',4'-dimethoxy-2-hydroxyacetophenone, and (**8**) (E)-8,11,12-trihydroxyoctadec-9-enoic acid.

First, we performed an unsupervised statistical analysis using principal component analysis (PCA) on SIMCA^®^ (Fig. 1a). The PCA showed good separation of the seven different fractions F1 to F7 (Fig. 1a). The two PCA components, 1 and 2, accounted for a combined 45% of the total variation. The highly polar fractions F1 (CAf1) to F3 (CAf3) were grouped to the right while the moderately polar fractions F4 (CAf4) to F7 (CAf7) were organised to the left of the PCA scores plot (Fig. 1a). For better resolution, supervised multivariate analysis was performed using orthogonal projections to latent structures Discriminant Analysis (OPLS-DA). The OPLS-DA scores plot was created, and it distinctly showed separation of active and inactive fractions (Fig. 1b). The quality of the OPLS-DA model was assessed by evaluation of the goodness of fit and predictive ability. The following parameters, R^2^X = 0.529, R^2^Y = 1, and Q^2^ = 0.991 were observed indicative of the OPLS-DA model to be a good fit with acceptable predictive capacity.

**Figure 2:**
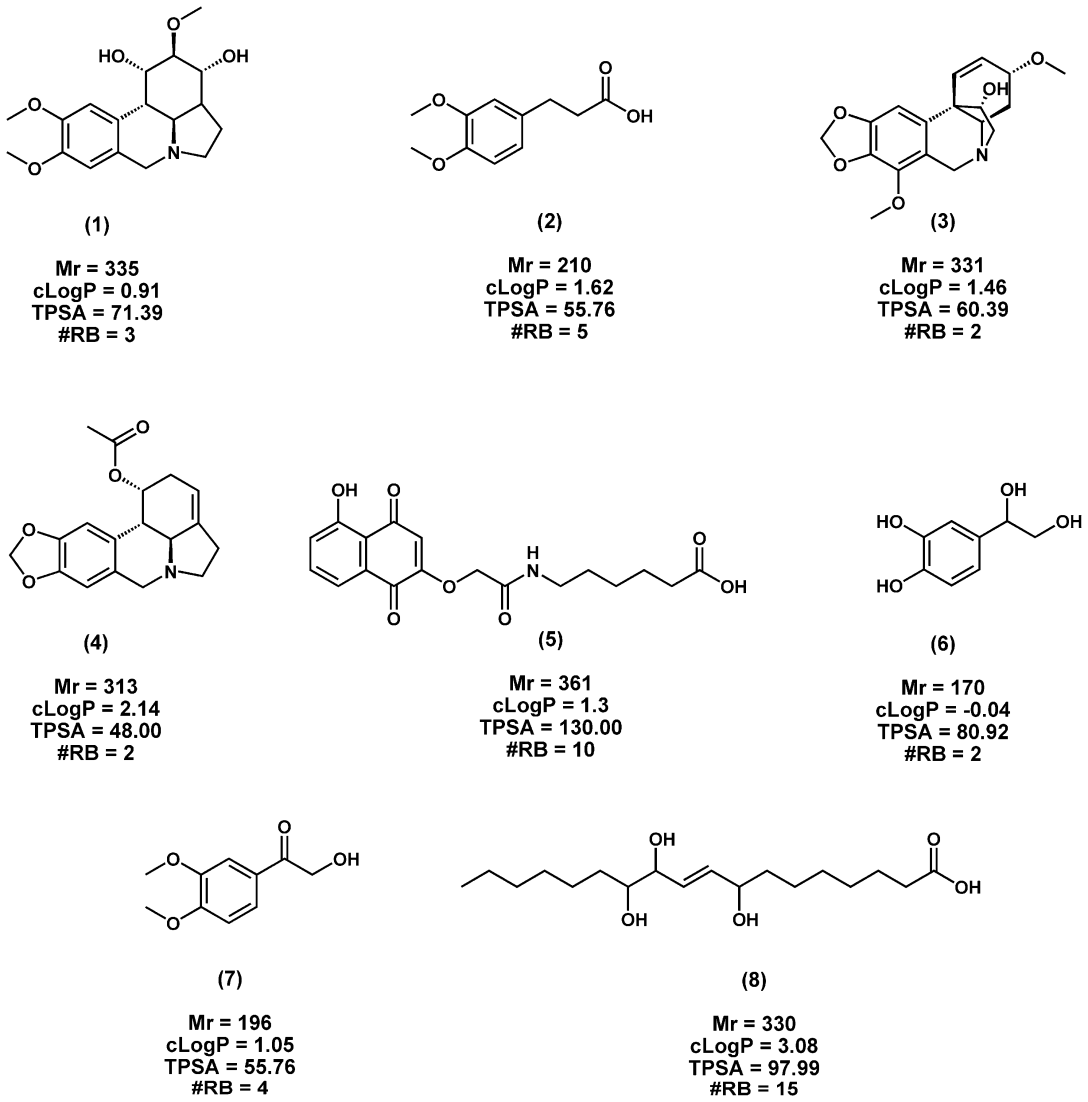
2D chemical representations of tentative compounds from *C. asiaticum* predicted to be active against *M. smegmatis* and *M. aurum*. (**1**) dihydronarcissidine, (**2**) 3,4-dimethoxyhydrocinnamic acid, (**3**) ambellin, (**4**) acetylcaranine, (**5**) 6-(2-(5-hydroxy-1,4-dioxo-1,4-dihydronaphthalen-2-yloxy)acetamido)hexanoic acid, (**6**) 3,4-dihydroxyphenylglycol, (**7**) 3',4'-dimethoxy-2-hydroxyacetophenone, and (**8**) (E)-8,11,12-trihydroxyoctadec-9-enoic acid. *Mr* molecular mass, *cLogP* consensus LogP, *TPSA* total polar surface area and *#RB* number of rotatable bonds. TPSA, cLogP and #RB were computed using the online SwissADME application^44^.

Having confidently shown that the OPLS-DA scores plot was able to satisfactorily separate the active and inactive fractions, we then generated its loadings S-plot (Fig. 1c). This loadings plot showed discriminating variables, each dot representing a feature (compound), unique between the two groups of fractions. Of most interest were the extreme discriminate features in the bottom left quadrant which are positively associated with antimycobacterial potency of the active fractions. This positive contribution to activity is further noted on the contributions plot and the respective VIP values for these variables (Fig. 1d). These discriminating features were then manually annotated based on analysis carried out on MarkerLynx (v 4.1) and published literature sources (Table 4 and Supplementary File F1 to F8)^33–40^. This process led to the tentative identification of eight annotated compounds, namely (**1**) dihydronarcissidine, (**2**) 3,4-dimethoxyhydrocinnamic acid, (**3**) ambellin, (**4**) acetylcaranine, (**5**) 6-(2-(5-Hydroxy-1,4-dioxo-1,4-dihydronaphthalen-2-yloxy)acetamido)hexanoic acid, (**6**) 3,4-dihydroxyphenylglycol, (**7**) 3',4'-dimethoxy-2-hydroxyacetophenone, and (**8**) (*E*)-8,11,12-trihydroxyoctadec-9-enoic acid (Table 4, Fig. 2). These eight compounds are potentially responsible for the potency against *M. smegmatis* and *M. aurum*. Using NPClassifier^41^, many of the compounds, namely **1**, **3**, and **4**, were assigned into the Pathway, Super Class and Class of Alkaloids, Tyrosine alkaloids and Amaryllidaceae alkaloids, respectively. Exceptions were compounds **5** (Polyketides, Naphthalenes and Naphthoquinones), **6** (Alkaloids, Tyrosine alkaloids and Phenylethylamines), **7** (Shikimates and Phenylpropanoids) and **8** (Fatty acids, Octadecanoids and Other Octadecanoids). Compound **2** was classified into Shikimates and Phenylpropanoids, Phenylpropanoids (C6-C3) and Cinnamic acids and derivatives based on structural similarity to cinnamic acid.

**Table 4:** Tentatively identified compounds from *C. asiaticum* predicted from the OPLS-DA loadings S-plot to be active against *M. smegmatis* and *M. aurum*.

S/N	Rt (min)	Observed mass (*m/z*)	Calculated mass (*m/z*)	Mass error (ppm)	Fit conf (%)	Adduct	MS/MS fragment ions (*m/z*)	Molecular formula	Tentative compound
1	5.80	336.1804	336.1811	−2.1	99.96	[M + H]^+^	308.0991 290.1598 261.1212 233.1255 167.0738	C_18_H_25_NO_5_	Dihydronarcissidine^33,42^
2	8.34	211.0954 233.0774	211.0970	3.8	96.35	[M + H]^+^ [M + Na]^+^	211.1042 193.0909 178.0701 165.1007	C_11_H_14_O_4_	3,4-Dimethoxyhydrocinnamic acid^38^
3	4.74	332.1489	332.1498	−2.7	98.98	[M + H]^+^	316.1086 300.1215 286.1120 274.1632 229.0959	C_18_H_21_NO_5_	Ambellin^34,42^
4	7.60	314.1381	314.1392	−3.5	99.97	[M + H]^+^	177.0609 145.0336 121.0700	C_18_H_19_NO_4_	Acetylcaranine^35^
5	6.28	362.1229	362.1240	−3.0	100	[M + H]^+^	344.1284 326.1143 310.1035 238.0959	C_18_H_19_NO_7_	6-(2-(5-Hydroxy-1,4-dioxo-1,4-dihydronaphthalen-2-yloxy)acetamido)hexanoic acid^36^
6	7.48	193.0484	193.0477	3.6	73.10	[M + Na]^+^	151.0453 110.0070	C_8_H_10_O_4_	3,4-Dihydroxyphenylglycol^33,39^
7	6.86	197.0802	197.0814	−6.1^a^	99.99	[M + H]^+^	179.0766 167.0394 151.0805	C_10_H_12_O_4_	3',4'-Dimethoxy-2-hydroxyacetophenone^33,40^
8	10.65	353.2296	353.2304	−2.5	76.21	[M + Na]^+^	313.0699 297.1361	C_18_H_34_O_5_	(E)-8,11,12-trihydroxyoctadec-9-enoic acid^37^

The compounds have been tentatively identified to confidence level 3^43^.

*Rt* retention time, *ppm* parts per million, *Fit conf* fit confidence. *m/z* mass to charge.

^a^Compound annotation outside the < 5 ppm mass error.

## Discussion

As the antimicrobial resistance phenomenon continues to render tuberculosis treatment regimens less effective, there is a need to discover and develop new therapeutics to turn the tide against this scourge. The aim of this study was to find new chemical scaffolds from which antimycobacterial drugs could be developed. To facilitate the process to identify potential antimycobacterial compounds from the potent plant species we employed UPLC-HRMS metabolomics integrated with multivariate analysis. This is a proven robust tool in the natural products drug discovery field for the identification of active compounds from different natural product sources including plants^31,32,45–51^. Apart from its reliability in predicting potent compounds, it also has the advantage of being less time consuming compared to the classical bioassay-guided approach. This metabolomics approach allowed us to identify nine potential antimycobacterial compounds from *C. asiaticum*.

**Figure 3:**
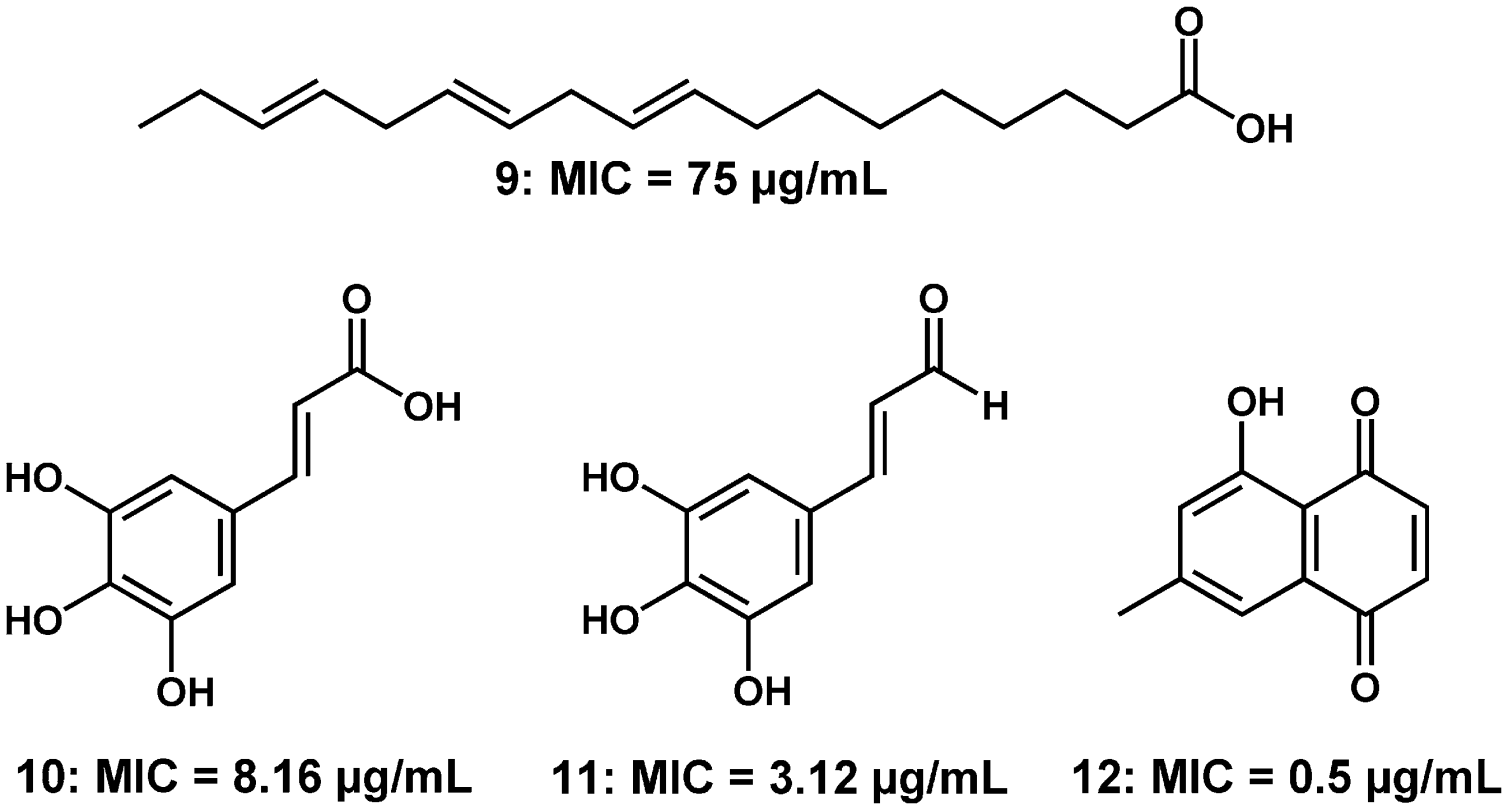
Activity of the fatty acid linoleic acid (**9**), cinnamic acid (**10)**, cinnamaldehyde (**13**) and 7-methyljuglone (**12**) against *M. tuberculosis *in vitro.

A preeminent indicator as to what kind of compound in *C. asiaticum* could be potent against *M. tuberculosis* emerged from the in vitro assays against this bacterium. The hint was the fact that activity against *M. tuberculosis* was only observed in serum free medium. The influence of culture medium supplements, particularly serum and albumin, is well documented within the tuberculosis drug discovery field^29^. The influence is by way of extensively binding to some molecules, especially those that are lipophilic. When serum binds tightly to lipophilic compounds it reduces their concentration in medium to levels unable to exert any effect on the target organism^52^. This hypothesis could hold true for compound **8** which had a noticeable high cLogP making it lipophilic. It is logical to assume this could have happened in serum supplemented media leading to loss of activity which was regained in serum free medium. In fact, a thorough literature search revealed that structurally similar compounds, namely fatty acids, have been demonstrated to have activity against *M. tuberculosis *in vitro^53^. Choi^53^ showed moderate activity of different isomers of linoleic acid and their conjugated forms against *M. tuberculosis* grown in Middlebrook 7H9 broth supplemented with 10% (v/v) oleic acid/albumin/dextrose/catalase enrichment and 0.05% (v/v) Tween 80. This included compound **9**, structurally closely related to **8**, with an MIC value of 75 µg/mL (Fig. 3)^53^. The mechanism of action of fatty acids has been postulated to be possible through causing membrane leakage by disrupting its integrity^54^. From a medicinal chemistry perspective, this compound has limitations such as the high number of rotatable bonds. This could make it highly flexible, allowing it to fit into many different active sites hence becoming toxic.

Cinnamic acids have received marked interest in the antimycobacterial drug discovery field^55–57^. Most notable is the study by Andrade-Ochoa et al.^58^ in which cinnamic acid (**10**, Fig. 3) was evaluated and shown to be potent against *M. tuberculosis* H37Rv (MIC = 8.16 µg/mL) and *M. bovis* AN5 (MIC = 7.29 µg/mL) strains cultured in Middlebrook 7H9 liquid media supplemented with 10% Middlebrook OADC enrichment, 0.05% Tween, and 0.2% Glycerol. Equally impressively potent was cinnamaldehyde (**11**, Fig. 3) with MIC values of 3.12 µg/mL and 12.5 µg/mL against *M. tuberculosis* H37Rv and *M. bovis* AN5, respectively^58^. Compound **2** is structurally unique in that it has two ether groups. This novelty in its structural architecture could endow it with improved physicochemical properties. This has been observed in the conversion of the alcohol functional moiety in dihydroartemisinin to a methyl ether functional group in artemether. The structural transformation resulted in a more potent and biological stable drug, i.e., artemether^59^. This makes it a worthwhile undertaking to isolate and investigate compound **2**. Moreover, beyond potency against *M. tuberculosis*, cinnamic acids have also been shown to enhance the activity of tuberculosis drugs^56,57^. Interestingly, the different geometric isomers show marked differences in potency against *M. tuberculosis.* The *cis*-cinnamic is more potent than the *trans*-cinnamic form with minimum bactericidal concentrations of 2.5 µg/mL and 300 µg/mL, respectively, against *M. tuberculosis*^56^. The *cis*-cinnamic also had more pronounced synergistic activity in enhancing potency of standard tuberculosis drugs against MDR *M. tuberculosis*^56^. This could be an interesting avenue to explore for compound **2** as well. The predicted ADME properties of compound **2** do make it an interesting prospect for medicinal chemistry development.

The Amaryllidaceae alkaloids have received minimal interest for their antimycobacterial activity. Maafi et al*.*^60^ previously screened a series of 9 Amaryllidaceae alkaloids against a non-pathogenic *M. tuberculosis* H37Ra ITM-M006710 (ATCC 9431) strain. The culture medium used for the assay was Middlebrook 7H9 broth enriched with 0.4% glycerol and 10% Middlebrook OADC growth supplement. All nine compounds, including the highly toxic lycorine, were inactive in vitro against *M. tuberculosis*. In the same study, the authors developed derivatives of the Amaryllidaceae alkaloids. Interestingly, only the derivatives with high lipophilicity (cLogP ranging from 4.2 to 5.99) were observed to be active against *M. tuberculosis,* including one which had an MIC value of 1.25 µg/mL. However, the selectivity index of these derivatives was low, something which future studies will need to improve on^60^.

An interesting candidate to have emerged from our study is compound **5**, a naphthoquinone. Despite the limited evaluation of this class of natural products, a study by van der Kooy et al*.*^61^ examined the activity of six naphthoquinones, isolated from a South African plant species *Euclea natalensis* (Ebenaceae), against *M. tuberculosis*. They used “enriched Middlebrook 7H9 broth base supplemented with bovine serum albumin, catalase, casein hydrolysate and ^14^C labelled substrate (palmitic acid) as a source of carbon.”^61^. Most of the compounds showed good activity with 7-methyljuglone (**12**) emerging as the most potent with an MIC of 0.5 µg/mL against *M. tuberculosis* (Fig. 3). Because of the structural similarity between **12** and menaquinone, a substrate of enzymes involved in the *Mycobacterial* electron transport chain, the authors suggested that the mechanism of action of this compound could be by negatively affecting this essential biological process in *M. tuberculosis*. This presumably could be by way of competitive inhibition. Given the structural resemblance of compound **5** to menaquinone, it is logical to hypothesise that this compound might possibly demonstrate this mechanism of action. This will be a subject of future research.

The distribution of plant families evaluated in the study was largely consistent with that reported by Sharma et al.^62^ who showed the Asteraceae to be the most represented family for antimycobacterial studies in their comprehensive systematic study. The reported activity of *C. asiaticum* reported here is the most prolific thus far compared to previous studies^25^. This clearly demonstrated the advantage of fractionation in improving activity. It was surprising to note the less pronounced activity of *A. afra* fractions. From a prior study, *A. afra* DCM extract was active against *M. aurum* (IC_50_ = 270 µg/mL) while, interestingly, its water and methanol extracts were inactive^24^. This suggested that compounds of medium polarity were most likely responsible for activity, given the fact that polar compounds in the highly polar solvents were inactive. The extract used in the current study was a DCM : MeOH extract while fractionation was largely carried out using a combined mixture of water and methanol with CAN : MeOH (50:50), a medium polar solvent, only used in the last fractionation step. This could explain the lack of activity as most likely the fractionation process failed to extract the medium polar compounds in the crude extract. Only fraction F7 was active with an MIC = 200 µg/mL against *M. aurum*.

## Conclusion

To advance antimycobacterial drug discovery from plant derived natural products, our collaborative study investigated African medicinal plants leading to the identification of a fraction of *C. asiaticum* which showed pronounced activity against *M. smegmatis* and *M. aurum*. This fraction was additionally moderately active against *M. tuberculosis*. UPLC-HRMS combined with multivariate analysis led to the prediction of 3 Amaryllidaceae alkaloids, a cinnamic acid derivative, a naphthoquinone and fatty acid as active constituents. To the best of our knowledge, none of these eight compounds have been experimentally evaluated for their activity against *M. tuberculosis,* making them promising prospects for continued studies. These natural compounds will be isolated and screened for their antimycobacterial activity in future studies.

## Materials and methods

### Plant collection

Twenty-nine of the plants used in the study were collected from the Biodiscovery Center, Department of Chemistry, University of Pretoria. This batch of plants consists of those previously collected and prepared by Clarkson et al*.*^63^, Fouche et al.^64^, Fouche et al.^65^, Moyo et al.^66^ and Mianda et al.^67^. *C. asiaticum* and *C. jagus* were collected in Ghana as per previous reports^25,68^ (Supplementary File Table S1 and voucher specimen numbers included). Permission to collect plant material was provided by relevant authorities in Ghana and South Africa. Collected plants were identified by botanists with respective voucher specimens deposited either at the South African National Biodiversity Institute (SANBI), H.G.W.J. Schweickerdt Herbarium of the University of Pretoria, and the herbarium of the Faculty of Pharmacy and Pharmaceutical Sciences, KNUST, Kumasi, Ghana (Supplementary File Table S1). Collected plants were identified by the following, Mr. J. Simpson, University of Pretoria^66^, Dr. Henry Sam^69^ and Mr. Clifford Asare^25,68^, Department of Herbal Medicine, Faculty of Pharmacy and Pharmaceutical Sciences, Kwame Nkrumah University of Science and Technology, Ghana, and Mr D Schuhardt, Mr E Nienaber, Mr Hans Vahrmeijer, Mr J Male, Mr FJ Potgieter, DJE Venter, Mr E Nienaber and JD Spies (all employed by Council for Scientific and Industrial Research at the time of collections and identifications done at South African Biodiversity Institute) and Mr JJ Meyer of the South African Biodiversity Institute. All experiments were carried out in accordance with relevant guidelines and regulations.

### Extraction and fractionation

A modified version by Invernizzi et al*.*^70^ of the National Cancer Institute (NCI) protocol by Thornburg et al*.*^71^ was used for the microextraction and fractionation of the plant samples. Dry plant material (7.2 g) was loaded into an extraction glass vessel^70^. A 50 mL 1:1 mixture of dichloromethane (DCM) : methanol (MeOH) was added to the vessel containing the plant material and sonicated in an ultrasonic bath for 1 h at a temperature of *ca.* 38ºC^70^. Following the extraction, the DCM : MeOH solution was subsequently drained, and collected into round bottom flasks, before carrying out a second extraction cycle with 50 mL MeOH. The extracts were combined and dried using an SP Genevac HT6 (Genevac Ltd., Ipswich, UK). A HypeSep C8 SPE cartridge (2 g/6 ml) was used to fractionate each extract into 7 fractions using a Gilson GX-241 ASPEC® liquid handler^70^. The DCM : MeOH extract (600 mg) was dissolved in 6 ml of a DCM : MeOH solution (liquid handling done manually) and adsorbed onto a cottonwool roll. The cottonwool roll with the crude extract was dried before being transferred to an empty prewashed 10 mL SPE cartridge^70^. Fractionation commenced as previously described Invernizzi et al.^70^: 19:1 (H_2_O : MeOH); 4:1 (H_2_O : MeOH); 3:2 (H_2_O : MeOH); 1:1 (H_2_O : MeOH); 2:3 (H_2_O : MeOH); 1:4 (H_2_O : MeOH) and 1:1 (ACN : MeOH) resulting in 7 fractions (F1 to F7) collected per each extract. The collected fractions were dried using an SP Genevac HT6. The resulting dried fractions and extracts were dissolved in 100% dimethyl sulphoxide (DMSO) to a stock solution of 20 mg/mL and stored in a freezer at 4ºC prior to screening.

### Antimycobacterial screening

#### In vitro* M. smegmatis* and *M. aurum* screening

The MIC of fractions and the reference antibiotic was measured using the previously established high-throughput spot culture growth inhibition assay (HT-SPOTi) approach^26^. In summary, autoclaved Middlebrook 7H10 agar with 0.5% glycerol was used. Agar was treated with 10% (v/v) oleic albumin dextrose and catalase (OADC). To achieve a larger range of concentration, the extracts and fractions were serially diluted with 0.3% DMSO in a PCR half-skirted 96-well plate.

Mycobacterial strains (*M. smegmatis* (NCTC 8159, ATCC 19420) and *M. aurum* (NCTC 10437, ATCC 23366) were inoculated in Falcon tubes with 10 mL MB7H10 and incubated at 37ºC for 18–24 h. As previously reported by Danquah et al*.*^26^ and Ofori et al*.*^25^, 2 µL of extracts, fractions and positive control drug, rifamycin, were transferred from a PCR half-skirted plate into a 96-well plate. Prepared agar (200 µL) was added to each well of the plate containing the already dispensed fractions. Each well of the 96-well plate received 2 µL of the bacterial suspension containing *ca.* 1 × 10^6^ cfu/mL. The bacterial spot suspension was absorbed into the agar over a 5-min period. The plates were sealed with parafilm, wrapped with aluminum foil, and incubated at 37 °C for 18–24 h. Wells with no drug served as growth control. After incubation, plate was observed, and the minimum inhibitory (MIC) concentration determined. Assays were carried out in technical duplicates.

### In vitro* M. tuberculosis* assay

Antimycobacterial assays were outsourced to the H3D Center, University of Cape Town, South Africa^27^. The MIC, i.e., concentration required to inhibit 90% of the culture of *M. tuberculosis* H37RvMA ATCC 27294, was determined using the slightly microplate Alamar blue assay previously described by Franzblau et al*.*^28^. The crude extract and fraction F5 were evaluated in three media compositions as follows: (1) 7H9_ADC_GLU_TW: Middlebrook 7H9 media (Difco) supplemented with 0.2% Glucose, Middlebrook albumin-dextrose- catalase (ADC) enrichment (Difco) and 0.05% Tween 80, (2) 7H9_ADC_GLU_TX: Middlebrook 7H9 media (Difco) supplemented with 0.2% Glucose, Middlebrook albumin-dextrose- catalase (ADC) enrichment (Difco) and 0.05% Tyloxapol and (3) 7H9_CAS_GLU_TX: Middlebrook 7H9 media (Difco) supplemented with 0.4% Glucose, 0.03% Casitone (Gibco Bacto) and 0.05% Tyloxapol.

The test samples were received as liquid stocks at concentrations of 20 mg/mL and 16.7 mg/mL for the crude extract of *C. asiaticum* and its fraction F5, respectively. Two-fold serial dilutions were prepared in growth medium in 96-well round bottom plates. A concentration range of 125–0.244 µg/mL and 104.37–0.203 µg/mL was investigated for each sample respectively. Isoniazid and moxifloxacin were included as the positive control drugs in all experiments. A maximum inhibition control (rifamycin at 0.15 µM) and a minimum inhibition control (0.625% DMSO) were included on each test plate.

The culture of *M. tuberculosis* H37RvMA^72^, in 7H9_ADC_GLU_TW, was grown to an optical density (OD600) of 0.5–0.7 and diluted down to OD600 of 0.001 in each medium. 50 µL of the diluted culture (*ca.* 1 × 10^4^ bacilli) was added to each well of each test plate, for a final volume of 100 µL per well. Each plant sample was tested in technical duplicate. The assay plates were incubated at 37 °C with 5% CO_2_ and humidification for 7 days. One tenth of the assay volume of resazurin (0.2 mg/mL prepared in phosphate buffered saline) was then added to each well of the assay plate and re-incubated for 24 h. The relative fluorescence units (RFU) (excitation 540 nm; emission 590 nm) of each well was measured using a SpectraMax iD3-3130 plate reader at day 8 (Molecular Devices Corporation, CA, USA).

Data analysis was performed using the built-in Spectramax protocol. Briefly, raw RFU data was normalised to the minimum and maximum inhibition controls to generate a dose response curve (% inhibition), using the Levenberg–Marquardt damped least-squares method, from which the MIC is calculated using the 4-parameter curve fit protocol. The lowest concentration of drug that inhibits 90% of growth of the bacterial population was interpreted to be the MIC.

#### UPLC-HRMS instrumentation chromatographic and MS conditions

Chromatographic analysis was performed on a Waters Aquity UPLC and system (Waters Inc., MA, USA). The SPE-fractions were dissolved in MeOH to a concentration of 1500 ppm (1.5 mg/mL) following which they were centrifuged for 30 s using a Mini Centrifuge (BOECO, Germany) to completely dissolve the fractions. The dissolved fractions (5 µL per injection) were run using a Waters ACQUITY UPLC^®^ BEH C_18_ (2.1 mm × 100 mm, 1.7 µm) column (Waters Inc., MA, USA). The mobile gradient phase consisted of two solvents, namely A = H_2_O + 0.1% formic acid and B = MeOH + 0.1% formic acid, applied in gradient mode as follows, 0 min 3% B, 1 min 3% B, 14 min 100% B, 17 min 100% B, 17.5 min 3% B, 20 min 3% B. the flow rate and column temperature were held constant throughout the runs at 0.3 mL/min and 50 °C. The separated compounds were tentatively identified using a Xevo^®^ G2 QTof mass spectrometer, run in electrospray ionization positive (ESI^+^) mode, coupled to an ACQUITY QDa mass detector (Waters Inc., MA, USA). The system was operated with MassLynx™ (v 4.1) software (Waters Inc., MA, USA) for data independent acquisition. The source conditions were as follows: the capillary voltage for ESI was 2.8 kV ESI^+^. The source temperature was set at 120 °C, the sampling cone voltage at 30 V, extraction cone voltage at 4.0 V and cone gas (nitrogen) flow at 20 L/h. The desolvation temperature was set at 350 °C with a desolvation gas (nitrogen) flow rate of 600.0 L/h. Fragmentation was performed using high energy collision induced dissociation (CID). The fragmentation energy was set at 2 V and 3 V for the trap and collision energy, respectively. The ramping was set from 3 to 4 V and 20 to 40 V for the trap and transfer collision energy, respectively. Mass spectral scans were collected every 0.3 s. Leucine enkephalin (m/z 555.2693, 2 ng/µL) was used as an internal lockmass standard with direct infusion at a flow rate of 3 µL/min. The raw data was collected in the form of a continuous profile. Mass to charge ratios (*m/z*) between 50 and 1 200 Da were recorded. Compound annotation was carried out by generating the respective molecular formula, Fit Confidence %, accurate mass and fragmentation pattern on MassLynx (v 4.1) and comparing them with published data. Discovery setting tolerance was set to 5 ppm for *m/z*, 10% for i-Fit confidence and MassFragment software was used for fragment matching. Annotation of compounds was carried out to Confidence Level 3^43^. The physicochemical properties of the compounds were computed online using SwissADME application^44^.

### Data processing and chemometrics analysis 

Raw mass spectral data was processed using MarkerLynx XS Method Editor (v 4.1) (Waters Inc., MA, USA) and carried using the parameters indicated on Table 5. Features were generated using a marker tolerance of 0.05 Da for *m/z* and ± 0.2 min for RT. The resulting Data matrix sheet, which included peak intensities, retention time, *m/z* values was exported as a Microsoft Excel CSV sheet. The Excel sheet was used an input file on SIMCA^®^-P (v 17) (Sartorius Stedim Data Analytics AB, Umeå, Sweden) for principal component analysis (PCA) and orthogonal projections to latent structures Discriminant Analysis (OPLS-DA) multivariate analysis. The PCA and OPLS-DA models were constructed of Pareto Scale transformed data. For this analysis, active SPE-fractions were classified as those with an MIC ≤ 30 µg/mL. Results generated by the PCA and OPLS-DA models were visualised as scores and loadings plots. Discriminate variables of significance were noted from the S-plot, their corresponding variable importance in the projection (VIP) values (> 1) and contribution plot generated from the OPLS-DA model.

**Table 5:** Method parameters for UPLC-HRMS data processing on MarkerLynx XS Method Editor (v 4.1).

Property	Value
Function	1
Analysis type	Peak detection
Initial retention time	0.00
Final retention time	20.00
Low mass	50
High mass	1200
Use relative retention time?	No
Peak width at 5% height (sec)	1.00
Peak-to-peak baseline noise	0.00
Apply smoothing?	No
Marker intensity threshold (counts)	10
Mass window (Da)	0.05
Retention time window (min)	0.20
Noise elimination level	0.00
Deisotope data?	No
Replicate % minimum	0.00

### Supplementary Information

Supplementary Information 1.

Supplementary Information 2.

Supplementary Information 3.

## Data Availability

The datasets generated in this study are available from the corresponding author on reasonable request.
