# Supplementary material for: Investigation of the antimycobacterial activity of African medicinal plants combined with chemometric analysis to identify potential leads
Source: Sci Rep. 2024 Jun 25;14:14660. doi: 10.1038/s41598-024-65369-7 (PMC11199645; doi:10.1038/s41598-024-65369-7)
Supplement: Supplementary file 1 — Supplementary Information 1. [file 41598_2024_65369_MOESM1_ESM.docx]

Supporting Information

**Investigation of the antimycobacterial activity of African medicinal plants combined with chemometric analysis to identify potential leads**

Phanankosi Moyo^1^^,a^, Michael Ofori^2,3a^, Olusola S. Bodede^1^, Madelien Wooding^1^, Ndivhuwo Kevin Khorommbi^1^, Lyndy J. McGaw^4^, Cynthia A. Danquah^2,^*, and Vinesh J. Maharaj^1,^*

^1^ Biodiscovery Center, Department of Chemistry, University of Pretoria, Pretoria 0028, South Africa

^2^Department of Pharmacology, Faculty of Pharmacy and Pharmaceutical Sciences, College of Health Sciences, Kwame Nkrumah University of Science and Technology, PMB, Kumasi Ghana.

^3^Department of pharmaceutical sciences, Dr Hilla Limann Technical University, Wa Ghana

^4^Phytomedicine Programme, Department of Paraclinical Sciences, Faculty of Veterinary Science, University of Pretoria, Private Bag X04, Onderstepoort, Pretoria 0110, South Africa

Phanankosi Moyo ([phanankosimoyo@gmail.com](mailto:phanankosimoyo@gmail.com))

Michael Ofori ([michof2825@gmail.com](mailto:michof2825@gmail.com))

Olusola Bodede ([olusolabodede@gmail.com](mailto:olusolabodede@gmail.com))

Madelien Wooding ([madelien.wooding@up.ac.za](mailto:madelien.wooding@up.ac.za))

Ndivhuwo Kevin Khorommbi ([khorombink@gmail.com](mailto:khorombink@gmail.com))

Lyndy J. McGaw ([lyndy.mcgaw@up.ac.za](mailto:lyndy.mcgaw@up.ac.za))

Cynthia Amaning Danquah ([cadanquah.pharm@knust.edu.gh](mailto:cadanquah.pharm@knust.edu.gh))

Vinesh Maharaj ([vinesh.maharaj@up.ac.za](mailto:vinesh.maharaj@up.ac.za))

*Corresponding author: Vinesh J. Maharaj (Natural Products Chemistry Analysis)

Tel: +27 (0824665466)

Email address: vinesh.maharaj@up.ac.za

Department of Chemistry

University of Pretoria

Private Bag x 20

Hatfield, 0028

South Africa

*Corresponding author: Cynthia A. Danquah (Antimycobacterial Analysis)

Tel: +233 (0265458216)

Email address: [cadanquah.pharm@knust.edu.gh](mailto:cadanquah.pharm@knust.edu.gh)

Department of Pharmacology

Faculty of Pharmacy and Pharmaceutical Sciences

College of Health Sciences

Kwame Nkrumah University of Science and Technology.

Kumasi

Ghana.

^a^ Contributed equally to this work.

Table S1: Plants species selected for *in vitro* antimycobacterial screening.

| **Plant species (Family)** | **Voucher number** | **Part** | **Local name*** | **Traditional use*** |
| --- | --- | --- | --- | --- |
| *Solanum aculeastrum* (Solanaceae) | DS03080 | L | Murulwa (Venda), Umthuma, itunga (Xhosa) and thola (Tswana). | Used for the treatment of different human and animal ailments [1]. Related species used for tuberculosis treatment [2]. |
| *Geranium robustum* (Geraniaceae) | EV00201 | L | Vrouebossie – amarabossie (Afrikaans) and ngope-sethsoha, tlako (Sotho) [3]. | Genera within the Geraniaceae family, including *Geranium*, and *Perlagonium* are used for treatment of several diseases including bladder infections, diarrhoea [4] and tuberculosis [5]. |
| *Terminalia phanerophlebia* (Combretaceae) | JM00243 | L | AmaNgwe-amnyama, amaNgwe-omphofu (Zulu) and mambonjwane (Swati). | Used for the treatment of many diseases including pneumonia [1]. Related species used for tuberculosis treatment [2]. |
| *Momordica cardiospermoides* (Cucurbitaceae) | HV00555 | WP | Inshubaba (Swati) and ntwe (Tswana). | Used traditionally for the treatment of different diseases [6, 7]. Related species used for tuberculosis treatment [2]. |
| *Burchellia bubalina* (Rubiaceae) | BP00936 | L | Thobankomo (Xhosa), Golwane (Zulu) and Hlosana (Swati). | The plant is widely used in folk medicine [8-10] and ethnoveterinary including for the treatment of heart water [11]. |
| *Catha edulis* (Celastraceae) | PRU 121392 | L | Umhlwazi (Zulu), iqgwaka (Xhosa), lehlatse, lewang, and molomomonate (Sepedi) [2]. | It is used for the treatment of common cold and respiratory of disorders [12] including tuberculosis [2] amongst others. |
| *Senegalia burkei* (Fabaceae) | JM00311 | L | Umkhaya wehlalahlathi, umbabampala (Zulu), umkhaya (Swati), mokgwa (Tswana), and munanga (Venda). | Used to treat eye and back pain [13]. Related species used for tuberculosis treatment [2]. |
| *Hedychium flavescens* (Zingiberaceae) | DS02802 | S | Wild ginger (English) [14]. | Used for ritual purposes generally and additionally for traditional medicinal use [14, 15]. |
|  |  | F |  |  |
| *Siphonochilus aethiopicus* (Zingiberaceae) | PRE 34817 | WP | Indungulo, isiphephetho (Zulu) and African ginger (English) [16]. | Used for the treatment of many disorders including tuberculosis [2], malaria, asthma, and inflammation [16, 17]. |
| *Leonotis leonurus* (Lamiaceae) | PRU 121393 | L | Wilde dagga (Afrikaans), umfincafincane, (isiXhosa), and utshwala-bezinyoni (isiZulu). | Its widely used in traditional medicine including for influenza, epilespsy, chest infection, tuberculosis and headaches [2, 18]. |
| *Salvia runcinata* (Lamiaceae) | JS00182 | L | Isicakathi [1]. | The plant is used as a disinfectant and for the treatment of hives [19], burns and sores [20]. |
| *Salvia africana-lutea* (Lamiaceae) | FP00851 | L | Ssand sage (English), bruinsalie, sandsalie, strandsalie, and geelblomsalie (Afrikaans). | Used for the treatment of tuberculosis, influenza, common cold fever amongst many other ailments [21]. |
| *Jatropha erythropoda* (Euphorbiaceae) | HV00572 | T | Rooikambroo (Afrikaans)^1^ and Thotamadi (Tswana and Sepedi) [22, 23]. | Plant is administered as an immune booster for HIV/AIDS management [22] and other sexual transmitted diseases [23]. Related species used for tuberculosis treatment [2]. |
| *Hypericum roeperianum* (Hypericaceae) | MM00136 | L | Isivumelelwane (Zulu) [1]. | Used for treatment of different disorders including diarrhoea, pain, indigestion and bacterial diseases [24]. |
| *Garcinia gerrardii* (Hypericaceae) | DS02273 | L | Bosgeelmelkhout (Afrikaans), umbande (Xhosa), isibinda, (Zulu) and sikhwelamkhala (Swati). | Used traditionally for the treatment of tuberculosis [2]. |
| *Ptaeroxylon obliquum* (Rutaceae) | DS02499 | L | Mogabaletswana (Sepedi) [2] and umThathi (Xhosa). | Used traditionally for the treatment of tuberculosis [2]. |

*Data collated from PlantZAfrica (<https://pza.sanbi.org/about>) which is provided by the South African National Biodiversity Institute (<https://www.sanbi.org/>). Additional information on plants has been acquired from other sources including published peer reviewed articles. Traditional uses provided are not exhaustive. ^1^<http://redlist.sanbi.org/species.php?species=576-6>. ^2^<https://www.herbgarden.co.za/mountainherb/herbinfo.php?id=215>.

Continued Table S1: Plants species selected for *in vitro* antimycobacterial screening.

| **Plant species (Family)** | **Voucher Specimen** | **Part** | **Local name*** | **Traditional use*** |
| --- | --- | --- | --- | --- |
| *Ekebergia capensis* (Meliaceae) | FP00739 | L | Cape ash, dogplum (English), essenhout (Afrikaans), umnyamatsi (Swati), nyamaru (Tswana). | Used to facilitate child birth, treat headaches, and skin diseases [25]. |
| *Turraea obtusifolia* (Meliaceae) | PRU 129518 | LF | Dima (Sepedi) [22]. | Used traditionally for the treatment of tuberculosis [2]. |
| *Drimia sp.* (Asparagaceae) | FP00938 | WP |  | Related species used for tuberculosis treatment [2]. |
| *Cyrtanthus mackenii* (Amaryllidaceae) | DS04216 | L | Ifafa lily (English), and impingizana encane empofu (Zulu) [1]. | Used for treatment of cough, headache, cystitis and leprosy [26]. |
|  |  | R |  |  |
| *Tulbaghia simmleri* (Amaryllidaceae) | FP01384 | R | Ssweet wild garlic, sweet garlic, fragrant tulbaghia (English), and soetwildeknoffel (Afrikaans). | Used for ornamental purposes. Related species used for tuberculosis treatment [2]. |
|  |  | L |  |  |
| *Crinum jagus* (Amaryllidaceae) | KNUST/HM/B002/2018 | L | Sukooko (Akan, Ghana) [27]. | Used for asthma and tuberculosis treatment [28]. |
|  |  | B |  |  |
| *Crinum asiaticum* (Amaryllidaceae) | KNUST/HM2020/B004 | L |  | Used to treat respiratory tract infections [29] and skin infections. Related species is used for tuberculosis treatment [28]. |
|  |  | B |  |  |
| *Crinum sp.* (Amaryllidaceae) | QM00150 | B |  | Related species is used for tuberculosis treatment [28]. |
| *Arctotis stoechadifolia* (Asteraceae) | FP00817 | L | Trailing arctotis (English), kusgousblom, bittergousblom, and witgousblom (Afrikaans). | Used to “clean kidneys” [30]. |
| *Eriocephalus africanus var. paniculatus* (Asteraceae) | FP00896 | WP | Wild rosemary (English), wilderoosmaryn, and kapokbos (Afrikaans). | Used for many diseases including coughs, cold, expectorant, and antimicrobial agent [31]. |
| *Helichrysum odoratissimum* (Asteraceae) | FP01629 | L | Kooigoed, kruie (Afrikaans) and imphepho (Xhosa and Zulu). | Used as an incense, urinary tract infections, fever [1, 32]. Related species used for tuberculosis treatment [2]. |
| *Artemisia annua* (Asteraceae) | Cultivated | L | Sweet wormwood (English)^2^. | Used for the treatment of fever, and malaria [33]. Related species used for tuberculosis treatment [2]. |
| *Artemisia afra* (Asteraceae) | PRU 121389 | L | African wormwood (English), wilde-als (Afrikaans), mhlonyane (isiZulu) and lengana (Tswana). | Used for tuberculosis treatment [2, 30] amongst a plethora of ailments [34] |
| *Gymnanthemum corymbosum* (Asteraceae) | HV00485 | L | Mountain vernonia (English) and uhlunguhlungu (Zulu). | Used to treat fever, and malaria [35]. |
| *Gymnanthemum myrianthum* (Asteraceae) | JM00170 | L | Uhluhlunga, umhlungahlunga (Zulu) and linyatselo (Swati). | Related species used for malaria, fever, skin wounds and diarrhoea amongst other ailments [36]. |

Supplementary Files F1 to F8 for used for the tentative annotation of compounds from *Crinum asiaticum* using MassLynx.


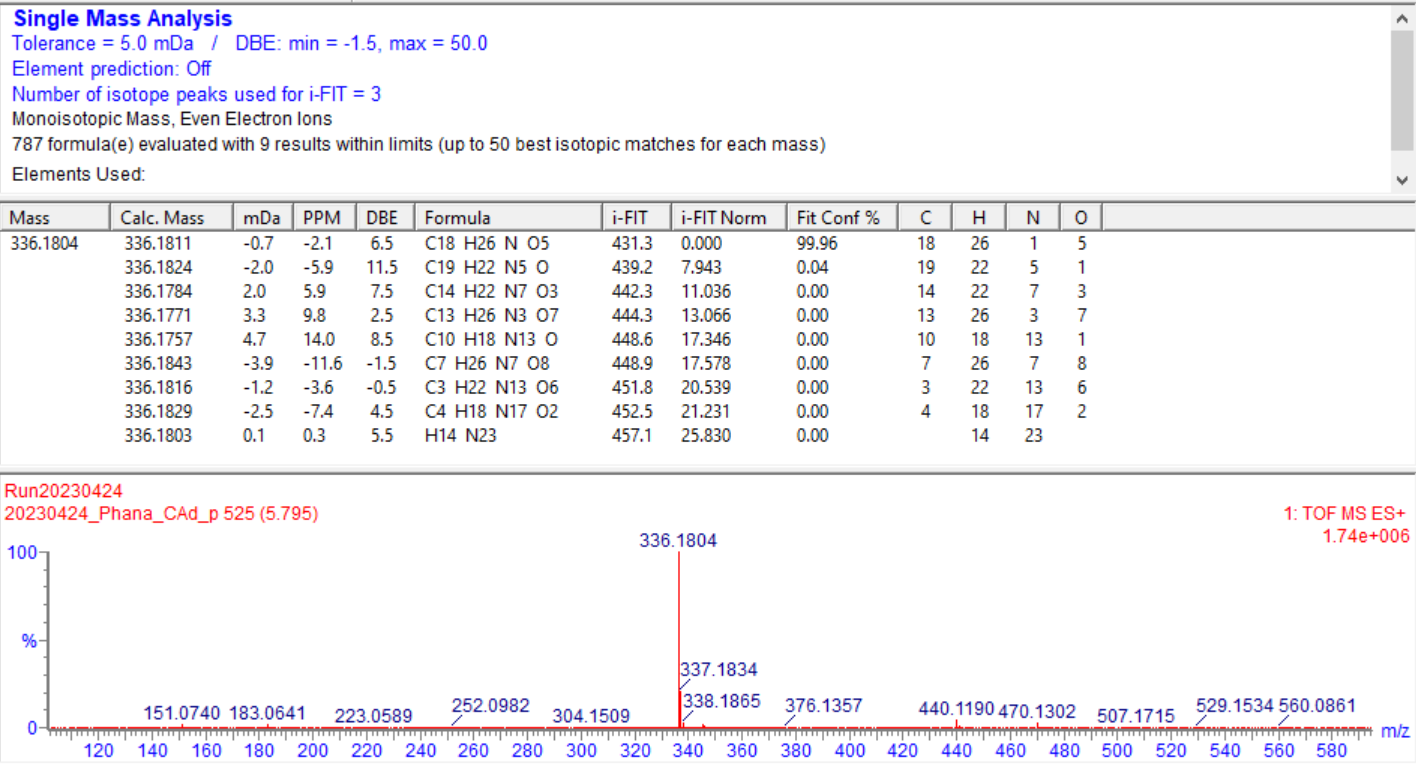


[M+H]^+^

Figure F1: Annotation for compound 1 on MassLynx


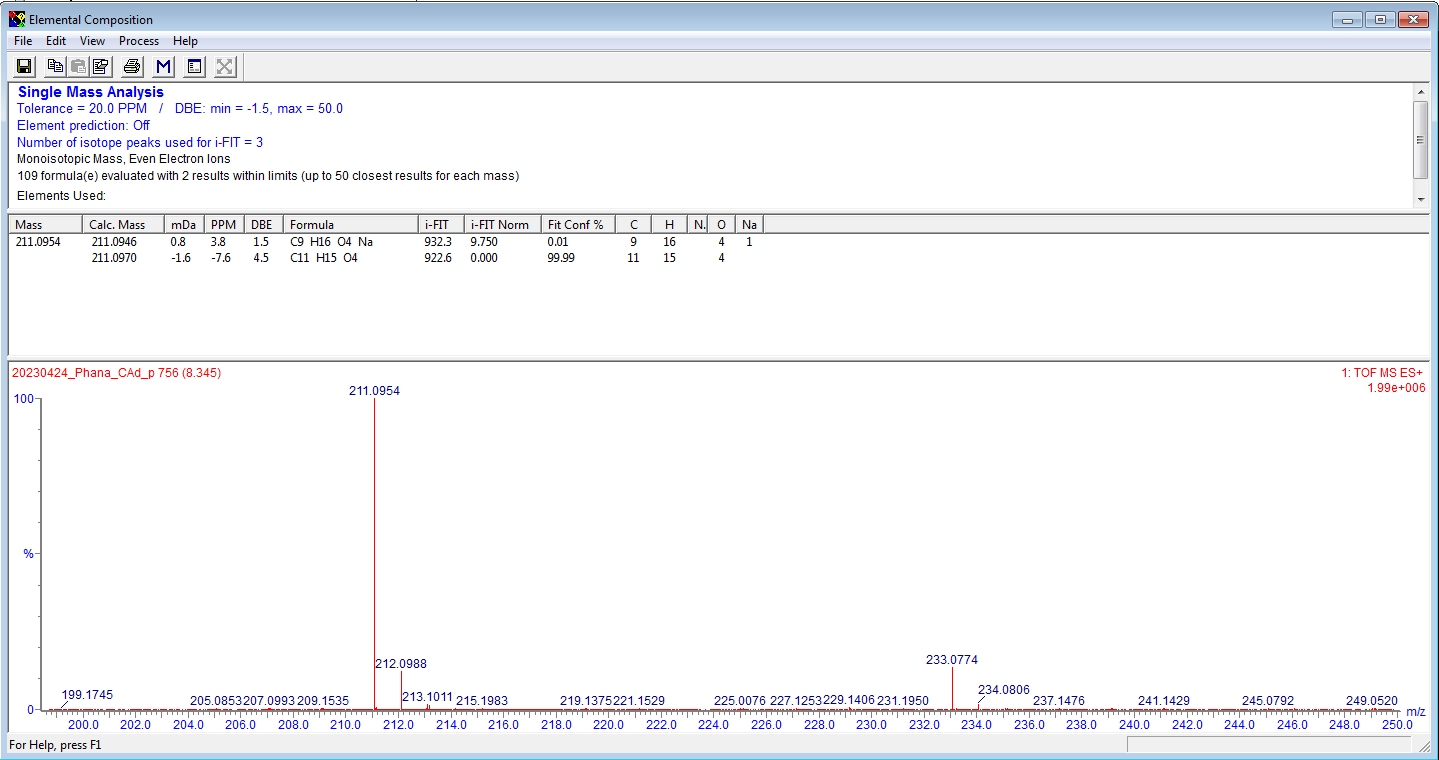


[M+Na]^+^

[M+H]^+^

Figure F2: Annotation for compound **2** on MassLynx


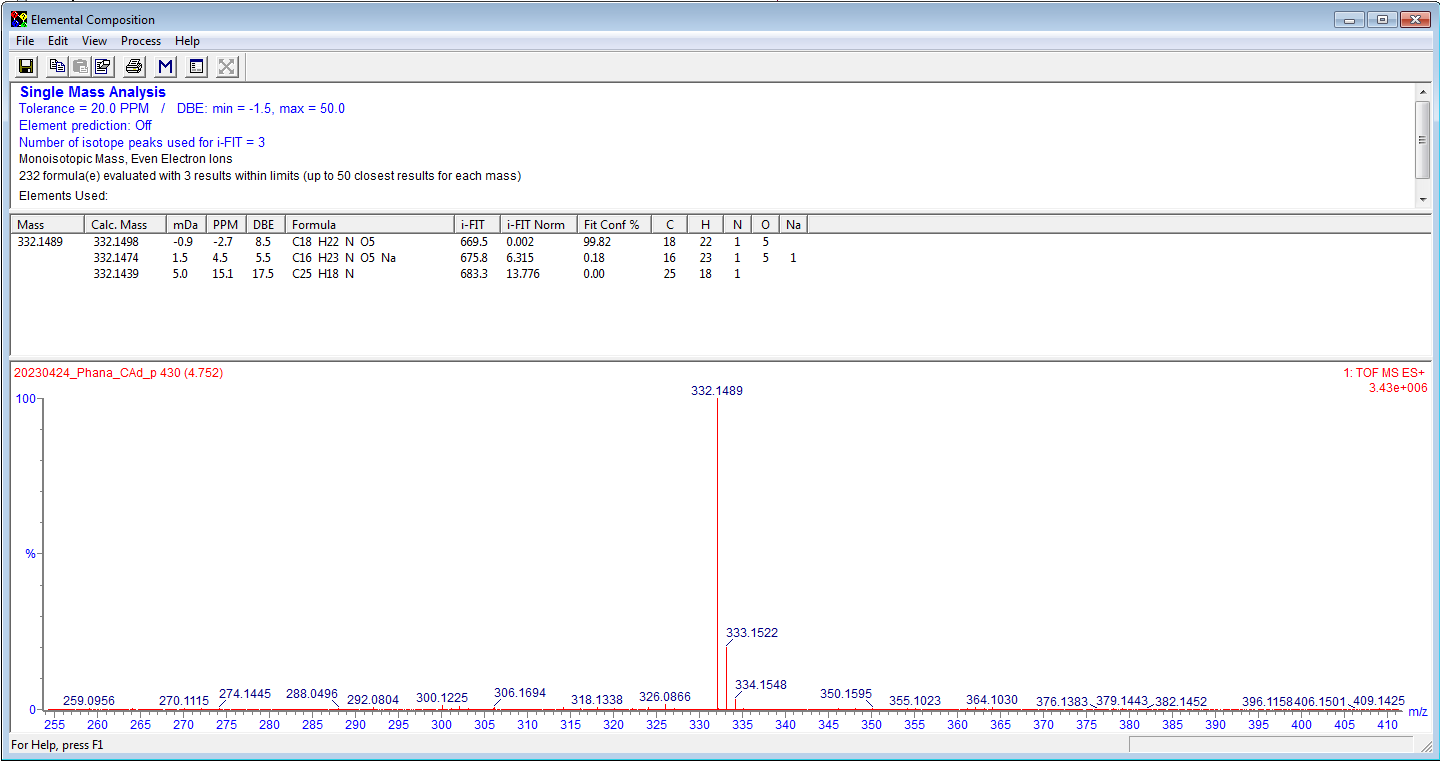


[M+H]^+^

Figure F3: Annotation for compound **3** on MassLynx


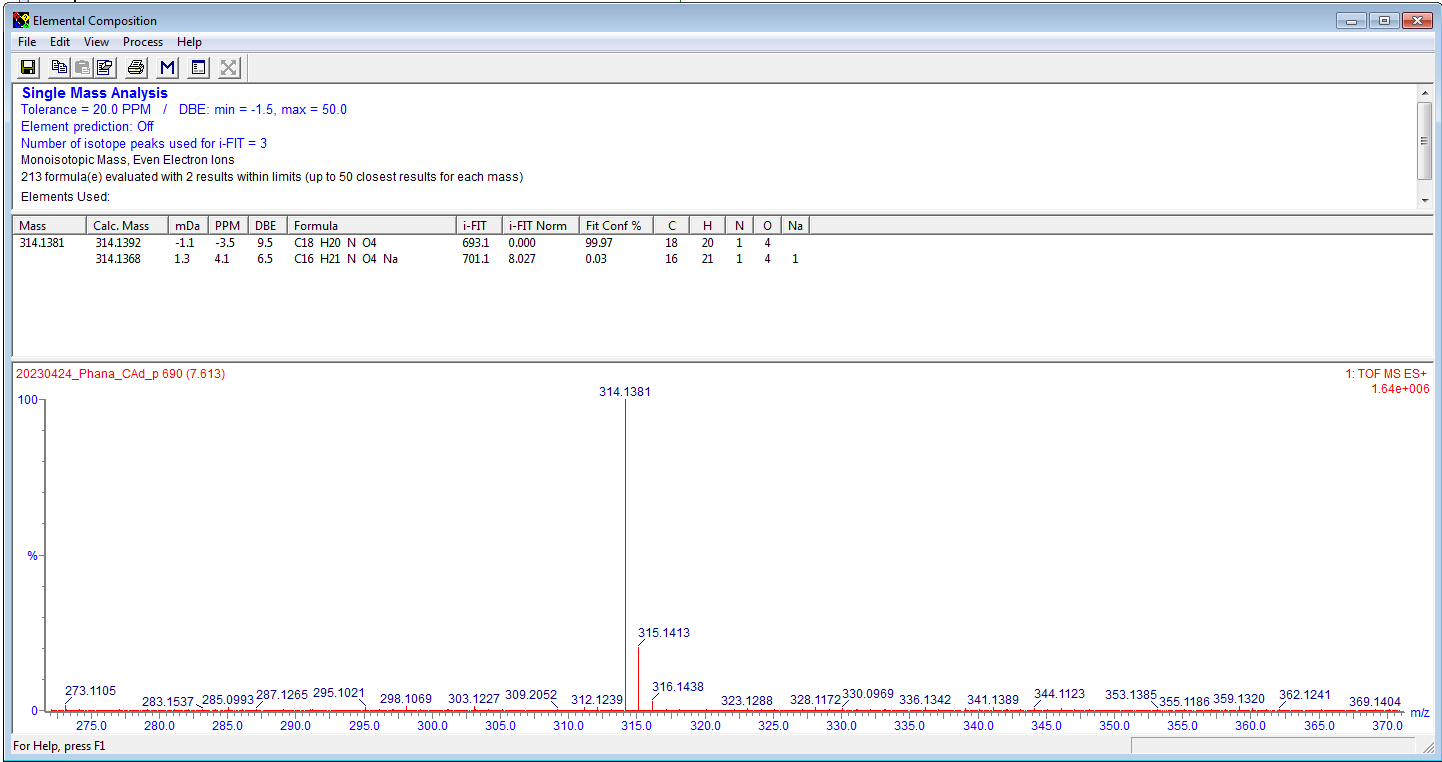


[M+H]^+^

Figure F4: Annotation for compound **4** on MassLynx


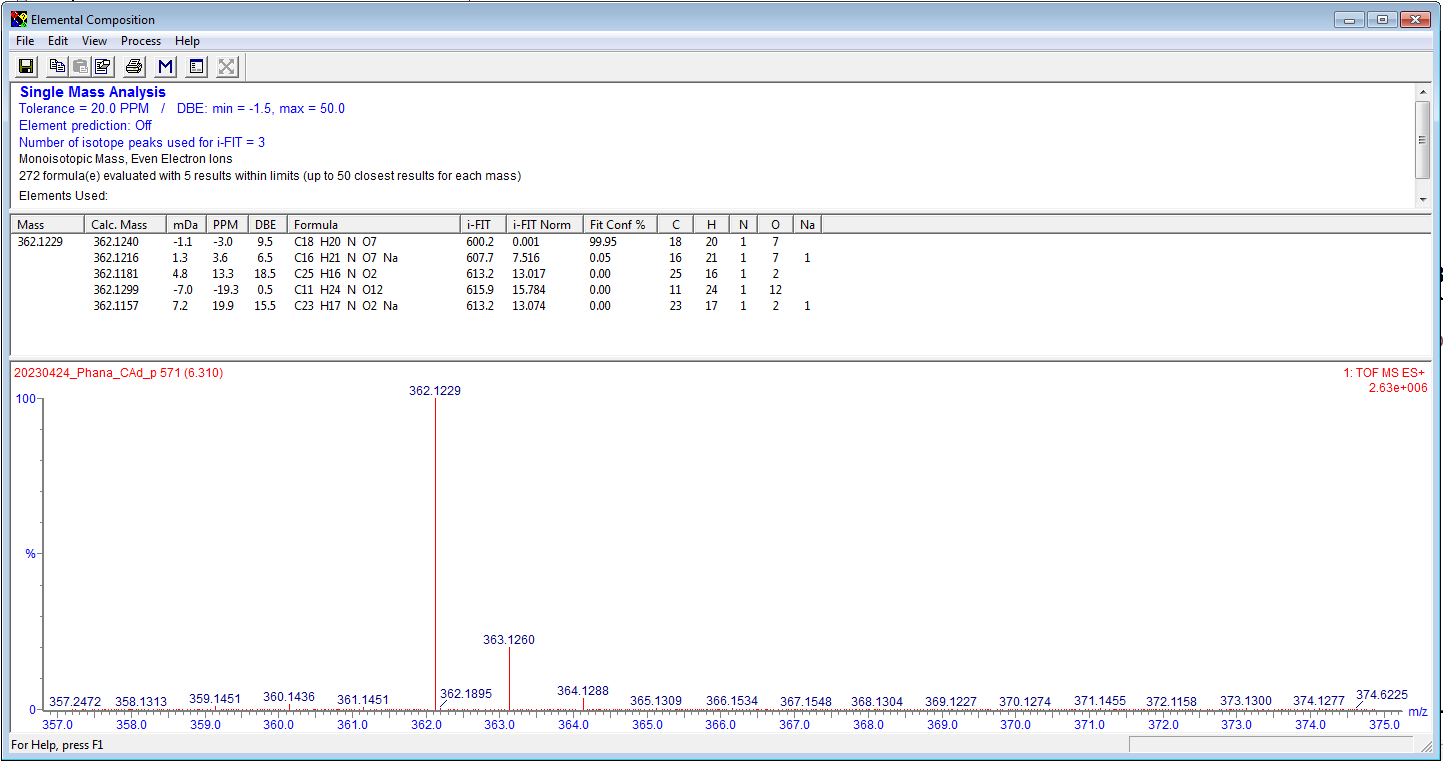


[M+H]^+^

Figure F5: Annotation for compound **5** on MassLynx


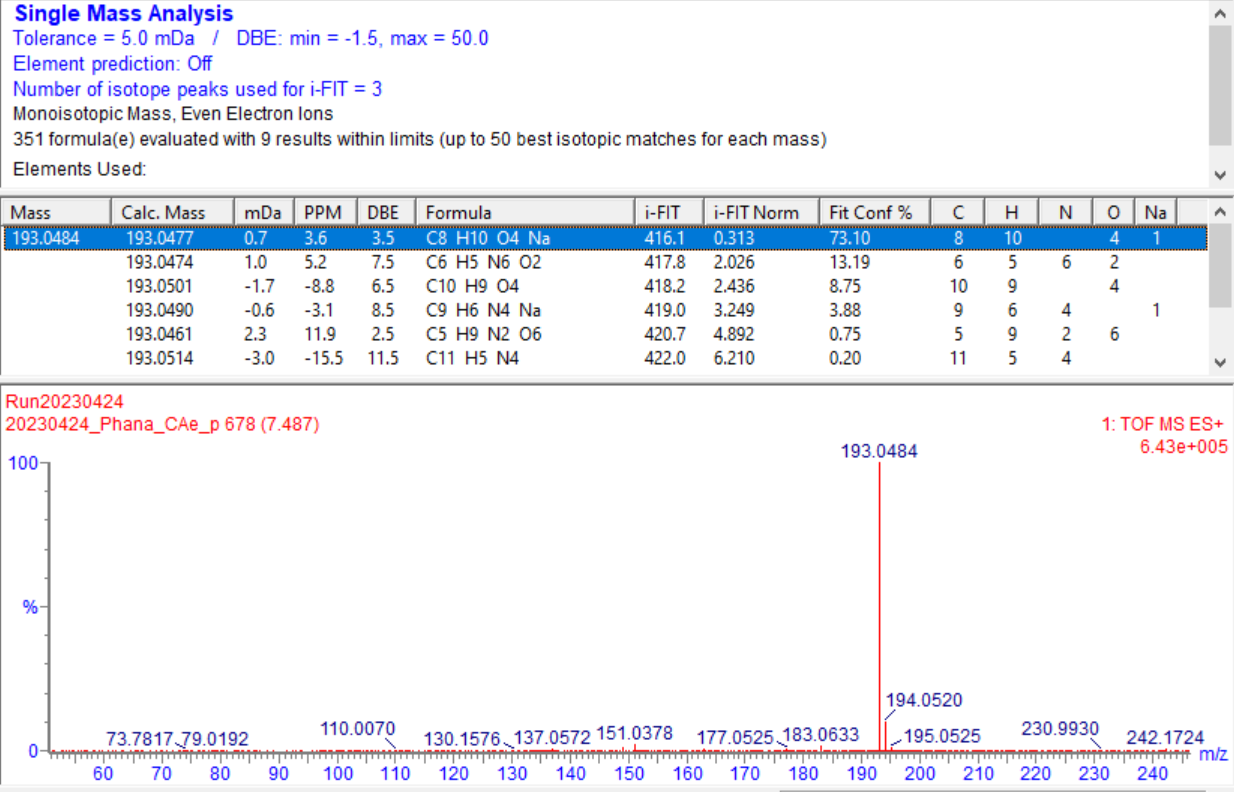


[M+H]^+^

Figure F6: Annotation for compound **6** on MassLynx


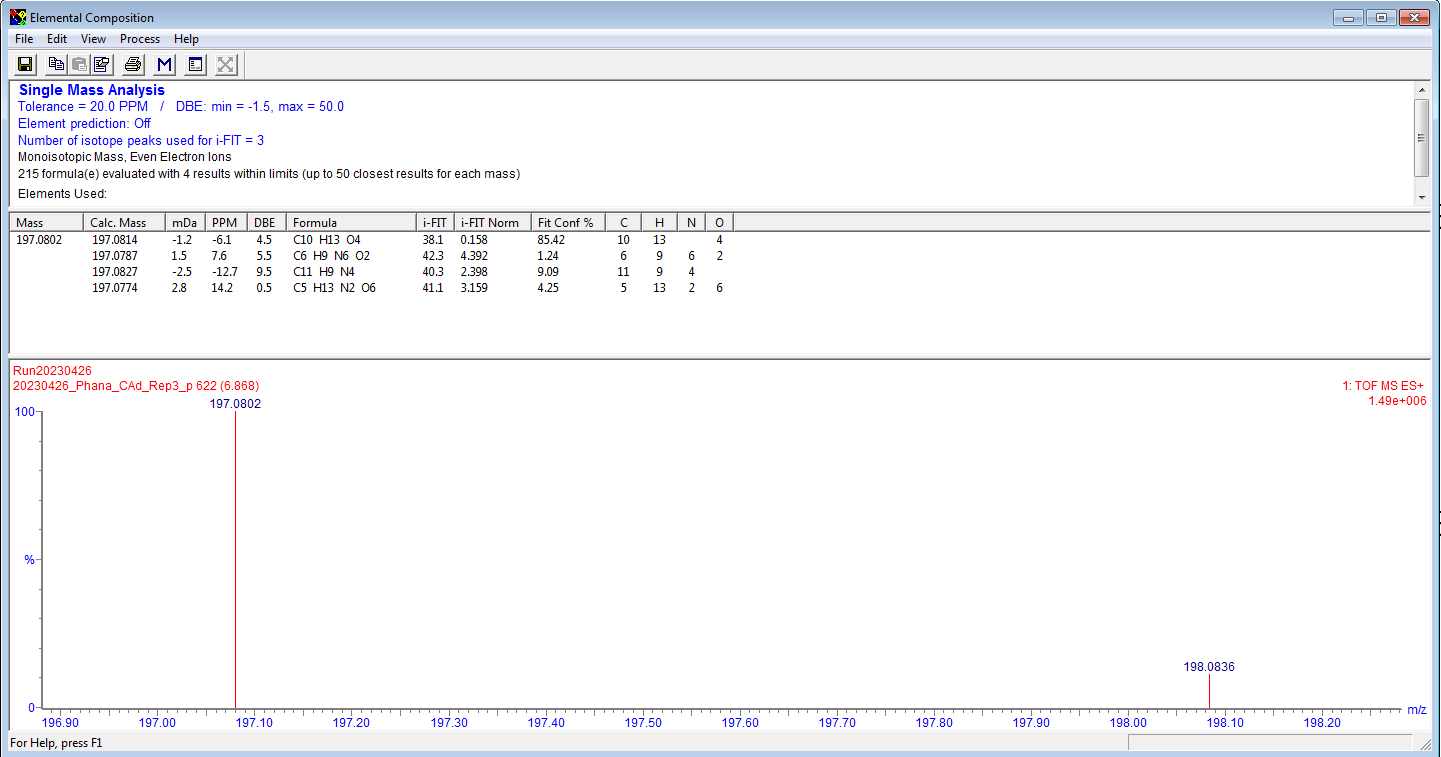


[M+H]^+^

Figure F7: Annotation for compound **7** on MassLynx


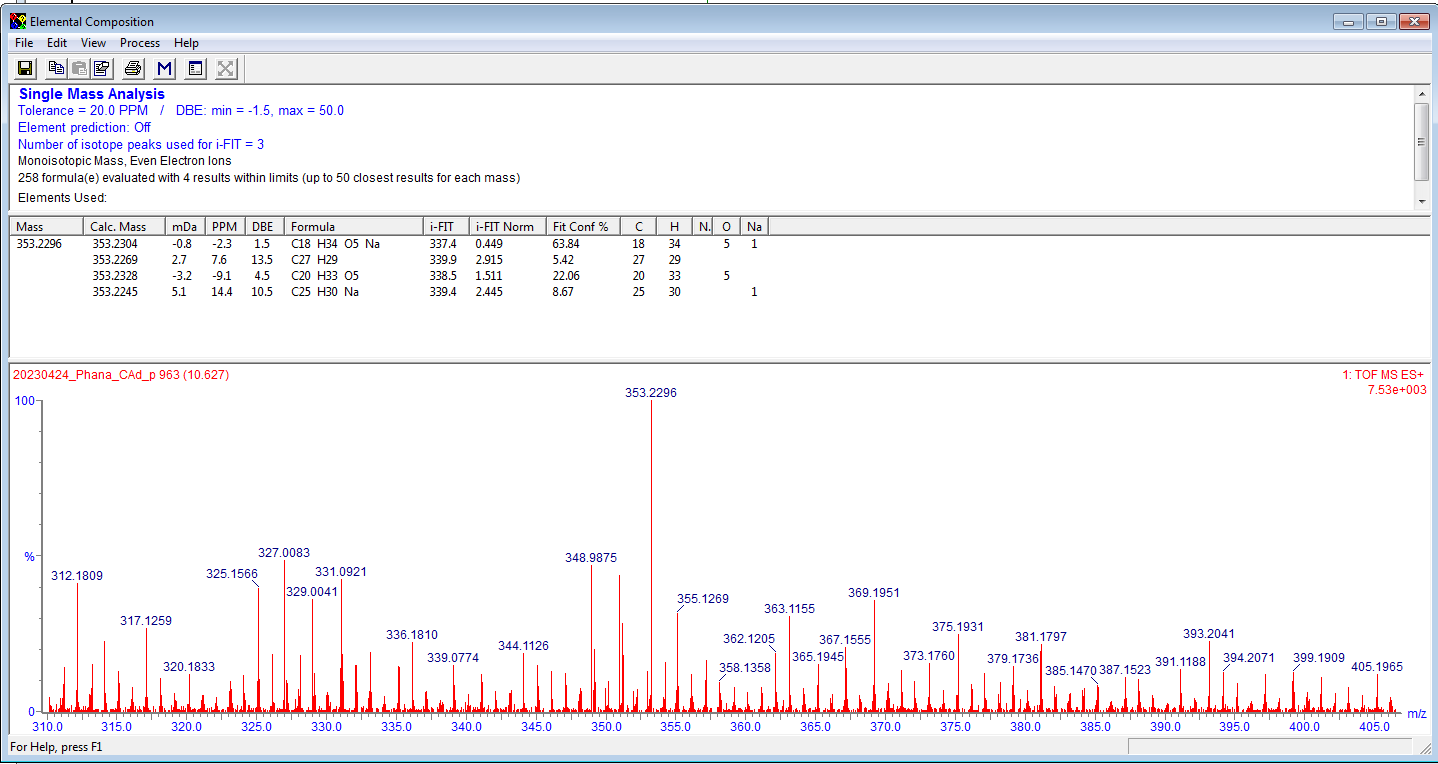


[M+H]^+^

Figure F8: Annotation for compound **8** on MassLynx

**7**

**5**

**4**

**3**

**1**

**Compound 2**

**Compound 6**

c

b

a

Figure F9: a) EIC (m/z 193.04) showing compound 6; b) EIC (m/z 233.07) showing compound 2; c) BPI chromatogram showing Compounds 1, 3, 4, 5 and 7.

**References**

1. Hutchings A: *Zulu medicinal plants: An inventory.* University of Natal press; 1996.

2. Semenya SS, Maroyi A: **Ethnobotanical survey of plants used by Bapedi traditional healers to treat tuberculosis and its opportunistic infections in the Limpopo Province, South Africa.** *South African Journal of Botany* 2019, **122:**401-421.

3. Babajide JO, Mabusela WT, Green I, Ameer F, Weitz F, Iwuoha EI: **Phytochemical screening and biological activity studies of five South African indigenous medicinal plants.** 2010.

4. Amabeoku GJ: **Antidiarrhoeal activity of Geranium incanum Burm. f. (Geraniaceae) leaf aqueous extract in mice.** *Journal of Ethnopharmacology* 2009, **123:**190-193.

5. Brendler T, van Wyk BE: **A historical, scientific and commercial perspective on the medicinal use of Pelargonium sidoides (Geraniaceae).** *Journal of Ethnopharmacology* 2008, **119:**420-433.

6. Ramabulana A-T, Petras D, Madala NE, Tugizimana F: **Metabolomics and molecular networking to characterize the chemical space of four Momordica plant species.** *Metabolites* 2021, **11:**763.

7. Muronga M, Quispe C, Tshikhudo PP, Msagati TAM, Mudau FN, Martorell M, Salehi B, Abdull Razis AF, Sunusi U, Kamal RM, Sharifi-Rad J: **Three Selected Edible Crops of the Genus Momordica as Potential Sources of Phytochemicals: Biochemical, Nutritional, and Medicinal Values.** *Frontiers in Pharmacology* 2021, **12**.

8. Amoo SO, Ndhlala AR, Finnie JF, Van Staden J: **Antibacterial, antifungal and anti-inflammatory properties of Burchellia bubalina.** *South African Journal of Botany* 2009, **75:**60-63.

9. Mhlongo LS, Van Wyk BE: **Zulu medicinal ethnobotany: new records from the Amandawe area of KwaZulu-Natal, South Africa.** *South African Journal of Botany* 2019, **122:**266-290.

10. Rana M, Rana M, Sharma D, Chauhan P: **Commonly used medicinal plants in tehsil Bangana, district una, Himachal Pradesh.** *Journal of Ayurvedic and Herbal Medicine* 2017, **3:**102-107.

11. Kambizi L: **Indigenous plants for ethnoveterinary uses in the Pondoland, South Africa.** In *XXIX International Horticultural Congress on Horticulture: Sustaining Lives, Livelihoods and Landscapes (IHC2014): V World 1125*2014: 309-314.

12. Ye S, Hu J, Liu Z, Liang M: **Progress and Research Trends on Catha edulis (Vahl) Endl. (Catha edulis): A Review and Bibliometric Analysis.** *Frontiers in Pharmacology* 2021, **12**.

13. Grace O, Prendergast H, Jäger A, Van Staden J, Van Wyk A: **Bark medicines used in traditional healthcare in KwaZulu-Natal, South Africa: An inventory.** *South African Journal of Botany* 2003, **69:**301-363.

14. Rojas-Sandoval J: **Hedychium gardnerianum (kahili ginger).** 2023.

15. Wu Z, Raven P, Hong D: **Flora of China; Missouri Botanical Garden Press: St.** *Louis, MO, USA* 1994, **2013**.

16. Adebayo SA, Amoo SO, Mokgehle SN, Aremu AO: **Ethnomedicinal uses, biological activities, phytochemistry and conservation of African ginger (Siphonochilus aethiopicus): A commercially important and endangered medicinal plant.** *J Ethnopharmacol* 2021, **266:**113459.

17. Seile BP, Bareetseng S, Koitsiwe MT, Aremu AO: **Indigenous Knowledge on the Uses, Sustainability and Conservation of African Ginger (Siphonochilus aethiopicus) among Two Communities in Mpumalanga Province, South Africa.** *Diversity* 2022, **14:**192.

18. Nsuala BN, Enslin G, Viljoen A: **“Wild cannabis”: A review of the traditional use and phytochemistry of Leonotis leonurus.** *Journal of Ethnopharmacology* 2015, **174:**520-539.

19. Kamatou GPP, Makunga NP, Ramogola WPN, Viljoen AM: **South African Salvia species: A review of biological activities and phytochemistry.** *Journal of Ethnopharmacology* 2008, **119:**664-672.

20. Rattray RD, Van Wyk B-E: **The Botanical, Chemical and Ethnobotanical Diversity of Southern African Lamiaceae.** *Molecules* 2021, **26:**3712.

21. Ezema CA, Aguchem RN, Aham EC, Ezeorba WFC, Okagu IU, Ezeorba TPC: **Salvia africana-lutea L.: a review of ethnobotany, phytochemistry, pharmacology applications and future prospects.** *Advances in Traditional Medicine* 2023.

22. Tietjen I, Gatonye T, Ngwenya BN, Namushe A, Simonambanga S, Muzila M, Mwimanzi P, Xiao J, Fedida D, Brumme ZL, et al: **Croton megalobotrys Müll Arg. and Vitex doniana (Sweet): Traditional medicinal plants in a three-step treatment regimen that inhibit in vitro replication of HIV-1.** *Journal of Ethnopharmacology* 2016, **191:**331-340.

23. Mathibela MK, Potgieter MJ, Tshikalange TE: **Medicinal plants used to manage sexually transmitted infections by Bapedi traditional health practitioners in the Blouberg area, South Africa.** *South African Journal of Botany* 2019, **122:**385-390.

24. Demgne OMF, Damen F, Fankam AG, Guefack MF, Wamba BEN, Nayim P, Mbaveng AT, Bitchagno GTM, Tapondjou LA, Penlap VB, et al: **Botanicals and phytochemicals from the bark of Hypericum roeperianum (Hypericaceae) had strong antibacterial activity and showed synergistic effects with antibiotics against multidrug-resistant bacteria expressing active efflux pumps.** *J Ethnopharmacol* 2021, **277:**114257.

25. Irungu BN, Orwa JA, Gruhonjic A, Fitzpatrick PA, Landberg G, Kimani F, Midiwo J, Erdélyi M, Yenesew A: **Constituents of the Roots and Leaves of Ekebergia capensis and Their Potential Antiplasmodial and Cytotoxic Activities.** *Molecules* 2014, **19:**14235-14246.

26. Kumari A, Baskaran P, Van Staden J: **In vitro propagation via organogenesis and embryogenesis of Cyrtanthus mackenii: a valuable threatened medicinal plant.** *Plant Cell, Tissue and Organ Culture (PCTOC)* 2017, **131:**407-415.

27. Minkah PAB, Danquah CA: **Anti-infective, anti-inflammatory and antipyretic activities of the bulb extracts of Crinum jagus (J. Thomps.) Dandy (Amaryllidaceae).** *Scientific African* 2021, **12:**e00723.

28. Idu M, Erhabor JO, Efijuemue HM: **Documentation on medicinal plants sold in markets in Abeokuta, Nigeria.** *Tropical Journal of Pharmaceutical Research* 2010, **9**.

29. Michael Ofori CAD, Paul Poku Sampene Ossei, Gilbrene Rahamani,, Isaac Newton Nugbemado, Peace Doe, Theophilus Kwesi Ninkyi: *Antitubercular activities of Crinum asiaticum bulb extract using aeroso-linduced Mycobacterium smegmatis in mice model.* Issue: 5; 2022.

30. Semenya S, Potgieter M, Tshisikhawe M: **Use, conservation and present availability status of ethnomedicinal plants of Matebele-Village in the Limpopo Province, South Africa.** *African Journal of Biotechnology* 2013, **12**.

31. Khalil N, Elhady SS, Diri RM, Fekry MI, Bishr M, Salama O, El-Zalabani SM: **Salicylic Acid Spraying Affects Secondary Metabolites and Radical Scavenging Capacity of Drought-Stressed Eriocephalus africanus L.** *Agronomy* 2022, **12:**2278.

32. Serabele K, Chen W, Combrinck S: **Chapter 10 - Helichrysum odoratissimum.** In *The South African Herbal Pharmacopoeia.* Edited by Viljoen A, Sandasi M, Fouche G, Combrinck S, Vermaak I: Academic Press; 2023: 247-258

33. Hien TT, White NJ: **Qinhaosu.** *The Lancet* 1993, **341:**603-608.

34. Liu NQ, Van der Kooy F, Verpoorte R: **Artemisia afra: A potential flagship for African medicinal plants?** *South African Journal of Botany* 2009, **75:**185-195.

35. Kizito IG, Mohammed K: **Phytochemical analysis, antimicrobial and antioxidant activities of leaf extract of Vernonia tigna Klatt (Asteraceae).** *World* 2022, **3:**016-023.

36. Vonia S, Hartati R, Insanu M: **In Vitro Alpha-Glucosidase Inhibitory Activity and the Isolation of Luteolin from the Flower of Gymnanthemum amygdalinum (Delile) Sch. Bip ex Walp.** *Molecules* 2022, **27:**2132.
